# The neuroimmune axis of Alzheimer’s disease

**DOI:** 10.1186/s13073-023-01155-w

**Published:** 2023-01-26

**Authors:** Mehdi Jorfi, Anna Maaser-Hecker, Rudolph E. Tanzi

**Affiliations:** 1 Genetics and Aging Research Unit, Department of Neurology, Mass General Institute for Neurodegenerative Disease, Massachusetts General Hospital, Charlestown, MA USA; 2 Harvard Medical School, Boston, MA USA; 3 McCance Center for Brain Health, Massachusetts General Hospital, Boston, MA USA

**Keywords:** Alzheimer’s disease, Heterogeneity, Immune system, β-amyloid, Neuroimmune

## Abstract

Alzheimer’s disease (AD) is a genetically complex and heterogeneous disorder with multifaceted neuropathological features, including β-amyloid plaques, neurofibrillary tangles, and neuroinflammation. Over the past decade, emerging evidence has implicated both beneficial and pathological roles for innate immune genes and immune cells, including peripheral immune cells such as T cells, which can infiltrate the brain and either ameliorate or exacerbate AD neuropathogenesis. These findings support a neuroimmune axis of AD, in which the interplay of adaptive and innate immune systems inside and outside the brain critically impacts the etiology and pathogenesis of AD. In this review, we discuss the complexities of AD neuropathology at the levels of genetics and cellular physiology, highlighting immune signaling pathways and genes associated with AD risk and interactions among both innate and adaptive immune cells in the AD brain. We emphasize the role of peripheral immune cells in AD and the mechanisms by which immune cells, such as T cells and monocytes, influence AD neuropathology, including microglial clearance of amyloid-β peptide, the key component of β-amyloid plaque cores, pro-inflammatory and cytotoxic activity of microglia, astrogliosis, and their interactions with the brain vasculature. Finally, we review the challenges and outlook for establishing immune-based therapies for treating and preventing AD.

## Background

Alzheimer’s disease (AD) is a neurodegenerative and genetically complex age-related dementia characterized by progressive memory loss. The pathogenesis of AD involves deposition of β-amyloid plaques, and formation of neurotoxic oligomers of the amyloid-β (Aβ) peptide. This results in neurofibrillary tangles (NFTs) made up of the hyperphosphorylated microtubule-associated protein, Tau (p-Tau), neuroinflammation, neuronal and synaptic loss, and, ultimately, onset of dementia [1–4]. Neuroimaging studies have revealed that β-amyloid plaques begin to deposit in the brain a decade or more before the onset of cognitive decline [5]. This indicates that therapeutics aimed at lowering Aβ, the key component of β-amyloid plaques, would be best used pre-symptomatically, preferably a decade or more before the propagation of AD pathologies. This form of prophylactic clinical strategy would be analogous to reducing future risk for heart disease by managing cholesterol levels [6].

β-amyloid deposition and tauopathy, as assessed by levels of Aβ species and p-Tau (p-Tau 181, 217, 231), respectively, in cerebrospinal fluid (CSF) and plasma as well as directly by positron emission tomography (PET), can be used to detect Aβ- and Tau-related neuropathology prior to the onset of cognitive impairment [7]. Recent studies have demonstrated that post-translational alterations of Tau also play a role in the rate of clinical AD progression [8] and variability in tauopathies across brain regions of patients [9]. Collectively, these studies suggest that AD is a heterogeneous neurodegenerative condition with respect to both clinical presentation and progression of AD pathology. Thus, it is important to consider how disease heterogeneity and the temporal order of AD neuropathological features impact target identification, the timing of treatment, and the development of therapeutics to reduce AD pathologies and treat cognitive symptoms.

While aging is the leading risk factor for the onset of AD, family history and genetic risk factors play the second most prominent role. Genetically, AD pathology is a complex and heterogeneous disorder, encompassing a spectrum of genetic effect sizes. They range from fully or highly penetrant mutations causing familial AD (in the AD risk genes, *APP*, *PSEN1*, and *PSEN2*) and the common *APOE* ε4 allele with the most substantial impact on the risk on sporadic AD to variants with relatively small effects on the risk identified in genome-wide associations studies (GWAS) [1, 2]. GWAS has implicated many genes with potential roles in adaptive and innate immune systems (Table 1), with *CD33* as the first of these genes shown to be associated with AD in a family-based GWAS [10]. This is followed by identifying other important AD-associated genes, including *TREM2*, *INPP5D*, *CLU*, *CR1*, *SPI1*, *ABCA7*, *EPHA1*, and the *MS4A* cluster [3, 10–14], with related functions to the immune system. *CLU* and *CR1*, for example, are well-established AD risk genes and components of the complement cascade. CR1 plays an important role in the activation of the complement system, mediates microglia activity, and promotes the phagocytosis of immune complexes, cellular debris, and Aβ [15–17]. *CR1*’s role in AD neuropathogenesis is still unknown. However, GWAS variants implicating that the *CR1* gene may lead to loss of function (LOF), a reduction of peripheral Aβ clearance by erythrocytes, and a dysregulation of the complement cascade, including effects on inflammation [16, 17]. Moreover, expression of the *CR1* gene in several cell types, such as erythrocytes, lymphocytes (T and B cells), and astrocytes, indicates that *CR1*’s mechanism of action on AD might be mediated through brain-resident cells and/or both peripheral immune cells and brain-specific cell types [18, 19]. Leveraging bispecific antibodies to simultaneously bind soluble Aβ and erythrocyte CR1 is suggested to rapidly decrease circulating Aβ as CR1-associated immune complexes [20]. This strategy might harbor the potential to subsequently prevent β-amyloid deposition in AD brains by an overall reduction of Aβ concentration in the bloodstream and other compartments [20]. Overall, the genetic heterogeneity of AD carries significant implications for drug development, which must be deeply considered in developing effective diagnostics and disease-modifying therapies for AD [21].

**Table 1:** Genome-wide significant AD-associated genes with potential roles in innate and adaptive immunity

**Chr**	**Gene**	**Immune-related function**	**References**
1	*ADAMTS4*	Immunomodulator	Jansen et al., 2019 [22]; Redondo-García et al., 2021 [23]
1	*AGRN*	Survival and function of monocytes	Mazzon et al., 2012 [24]
1	*CR1*	Immunity—e.g., microglial phagocytosis and clearance of complement opsonized molecules	Lambert et al., 2009 [25]; Borucki et al., 2020 [26]
1	*PSEN2*	Innate immune system	Agrawal et al., 2016 [27]; Nam et al., 2022 [28]; Fung et al., 2020 [29]; Mendez et al., 2017 [30]
1	*SORT1*	Monocytes and T cells	Bellenguez et al., 2022 [31]; Herda et al., 2012 [32]; Mortensen et al., 2014 [33]
2	*ADAM17*	T cell response	Lambrecht et al., 2018 [34]
2	*BIN1*	Pro-inflammatory response; endocytosis and phagocytosis	Seshadri et al., 2010 [35], Sudwarts et al., 2022 [36]
2	*FHL2*	Wound healing and inflammation	Nordhoff et al., 2012 [37]; Wixler et al., 2019 [38]
2	*INPP5D*	Immunity and microglia function	Efthymiou and Goate, 2017 [19]; Lambert et al., 2013 [39]
2	*SPRED2*	NK cells; cytokine/chemokine production	Itakura et al., 2017 [40]
2	*PRKD3*	Thymic selection during T cell development	Ishikawa et al., 2016 [41]
3	*IL17RD*	Cytokines	Brigas et al., 2021 [42]; Girondel et al., 2021 [43]
3	*MME*	Neutrophils	Schulte-Schrepping et al., 2020 [44]
4	*CLNK*	Immune cell-specific adaptors	Utting et al., 2004 [45]
4	*RHOH*	TCR signaling	Gu et al., 2006 [46]
4	*SCARB2*	IFN-I production and cholesterol regulation	Guo et al., 2015 [47]; Heybrock et al., 2019 [48]
5	*APC*	T cell migration	Mastrogiovanni et al., 2022 [49]
5	*HAVCR2*	Viral receptor; T cells	Zhai et al., 2021 [50]; Wightman et al., 2021 [51]
5	*HBEGF*	T cells	Macdonald et al., 2021 [52]
5	*MEF2C*	B cell proliferation, regulate microglia, and antigen presentation	Sao et al., 2018 [53]
5	*PFDN1/HBEGF*	Macrophage-mediated cellular proliferation	Higashiyama et al., 1991 [54]
5	*RASGEF1C*	Macrophages; microglia	Srinivasan et al., 2020 [55]
5	*TNIP1*	NF-κB regulation	G'Sell et al., 2016 [56]; Gurevich et al., 2011 [57]
6	*CD2AP*	T lymphocyte	Raju et al., 2018 [58]; Tao et al., 2019 [59]
6	*HLA-DRB5/* *HLA-DRB1*	Immunity—e.g., antigen presentation	Lambert et al., 2013 [39]; Lu et al., 2017 [60]
6	*TREM2*	Phagocytosis, and cellular metabolism in myeloid cells, microglia activation as well as binding to Aβ	Griciuc et al., 2019 [61]; Guerreiro et al., 2013 [13]; Jonsson et al., 2013 [14]; Kunkle et al., 2019 [62]; Bis et al., 2020 [63]; Sims et al., 2017 [64]
7	*EPHA1*	Immunity, BBB permeability, and immune cell trafficking	Ivanov et al., 2006 [65]; Chen et al., 2018 [66]
7	*IKZF1*	T/B cell dysregulation	Hoshino et al., 2022 [67]
7	*PILRA*	Immunoglobulin receptor; viral receptor	Agostini et al., 2019 [68]
7	*SEC61G*	Antigen presentation	Zehner et al., 2015 [69]
7	*TMEM106B*	Innate immune system	Rhinn et al., 2017 [70]
8	*CLU*	Immunity, binding to Aβ, and inhibition of complement system	Tschopp et al., 1993 [71]; Lambert et al., 2009 [25]; Zhao et al., 2015 [72]
8	*CTSB*	Immune cell infiltration	Ma et al., 2022 [73]; Ha et al., 2008 [74]
8	*PTK2B*	Spreading, migration, and function of immune cells	Okigaki et al., 2003 [75]; Lambert et al., 2013 [39]
8	*SHARPIN*	Neuroinflammation and NF-κB activation	Asanomi et al., 2019 [76]
9	*ABCA1*	Immune modulation; myeloid cells; dendritic cells	Zamanian-Daryoush et al., 2017 [77]; Westerterp et al., 2017 [78]
10	*BLNK*	B cell linker	Fu et al., 1998 [79]; Han et al., 2016 [80]
10	*ECHDC3*	NK, monocyte differentiation, cell infiltration	Zhao et al., 2022 [81]
10	*TSPAN14*	Mast cell function	Orinska et al., 2020 [82]
11	*MS4A*	Expressed in immune cells, TREM2 regulation, phagocytosis, regulation of complement system	Deming et al., 2019 [83]; Kuek et al., 2016 [84]
11	*PICALM*	Endocytosis and Aβ clearance	Zhao et al., 2015 [72]; Harold et al., 2009 [85]
11	*SORL1*	Microglia, monocyte migration, pro-inflammatory cytokines regulation and phagocytosis	Talbot et al., 2018 [86]; Knupp et al., 2020 [87]
11	*SPI1*	PU.1, altered microglia function	Jones et al., 2021 [88]
12	*TPCN1*	Antigen-presenting cells	He et al., 2020 [89]
14	*FERMT2*	Immune cell infiltration	Su et al., 2021 [90]
14	*IGHG1*	Antigen and immunoglobulin receptor binding activity	Lekhraj et al., 2022 [91]
14	*PSEN1*	Microglial hyperactivation	Lee et al., 2002 [92]
15	*ADAM10*	Immune cell function	Lambrecht et al., 2018 [34]
15	*CTSH*	Autoimmune inflammation, macrophages and phagocytosis, cytokines	Faraco et al., 2013 [93]; Conus et al., 2010 [94]; Zavašnik-Bergant et al., 2004 [95], Li et al., 2010 [96]
15	*IGF1R*	Stimulates regulatory T cells, autoimmunity	Bilbao et al., 2014 [97]; Andersson et al., 2018 [98]
15	*RORA*	Inflammatory response; lymphoid cell development; T cell survival; autoimmunity and chronic inflammatory response	Oh et al., 2019 [99]; Lo et al., 2016 [100]; Chi et al., 2021 [101]; Wang et al., 2021 [102]
15	*SPPL2A*	Catalyzes the intramembrane cleavage of the anchored fragment of shed TNF-α	Fluhrer et al., 2006 [103]
16	*IL34*	Proliferation, survival and differentiation of monocytes and macrophages	Foucher et al., 2013 [104]; Lin et al., 2008 [105]
16	*KAT8*	Viral immunity	Huai et al., 2019 [106]
16	*MAF*	Regulates IL-10	Gabryšová et al., 2018 [107]
16	*PLCG2*	Involved in multiple pathways in immune cells	Sims et al., 2017 [64]; Magno et al., 2021 [108]
16	*ZNF423*	B cell differentiation	Harder et al., 2013 [109]
17	*ABI3*	Immunity, regulation of actin polymerization, and microglia function	Sims et al., 2017 [64]; Satoh et al., 2017 [110]
17	*ACE*	Innate and adaptive immune system	Bernstein et al., 2018 [111]
17	*SCIMP*	MHC class II signaling transduction	Draber et al., 2011 [112]
17	*TSPOAP1*	Microglia-associated gene; IFN signaling	Bhatt et al., 2020 [113]
19	*ABCA7*	Microglia Aβ clearance and cholesterol metabolism	Hollingworth et al., 2011 [11]; Kim et al., 2006 [114]; Steinberg et al., 2015 [115]
19	*APOE*	Lipid metabolism; T cell activation	Saunders et al., 1993 [116]; Bonacina et al., 2018 [117]
19	*CD33*	Immunity, phagocytosis, and transmembrane receptor in myeloid cells	Bertram et al., 2008 [10]; Griciuc et al., 2013 [118]; Griciuc et al., 2019 [61]; Crocker et al., 2007 [119]
19	*LILRB2*	Immunoglobulin-like receptor; influence immune activation	Deng et al., 2021 [120]
20	*RBCK1*	Immunodeficiency disorders; LUBAC inhibits TNF-α signaling, dousing inflammation	Boisson et al., 2012 [121]; Bellenguez et al., 2022 [31]
20	*CASS4*	Eosinophil asthma response	Esnault et al., 2013 [122]
21	*ADAMTS1*	Immunomodulator	Rodríguez-Baena et al., 2018 [123]; Kunkle et al., 2019 [62]
21	*APP*	Antimicrobial peptide	Kumar et al., 2016 [124]; Eimer et al., 2018 [125]; Jonsson et al., 2012 [126]

The selected genes were implicated based on independent genetic loci with genome-wide significance in at least one GWAS in AD [12, 22, 25, 31, 39, 51, 85, 127–130] and a potential role in the immune system. *IFN-I* type I interferon; *NK cell* natural killer cell; *TCR* T cell receptor; *MHC* major histocompatibility complex; *NF-κB* nuclear factor kappa B; *LUBAC* linear ubiquitination assembly complex

**Fig. 1:**
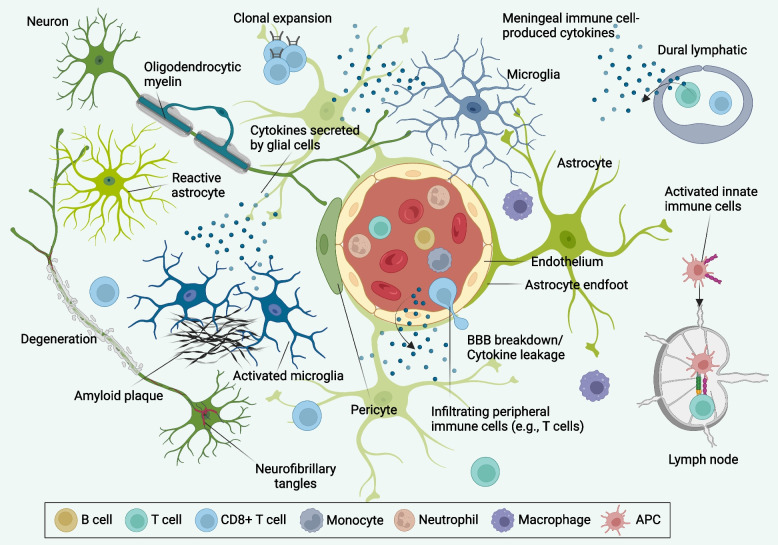
Neuroimmune interactions in AD neuropathology. AD is a heterogeneous and multifactorial complex neurodegenerative disease that is characterized by the abnormal aggregation of extracellular β-amyloid plaques and intracellular neurofibrillary tangles. This leads to neuronal cell death, synaptic degradation, and gliosis (microglia and astrocytes), further exacerbating neurodegeneration and ultimately leading to dementia. Under homeostatic conditions, microglia have a predominantly protective role, including phagocytosis and degradation of Aβ, secretion of anti-inflammatory cytokines, and neural network remodeling. However, excessive β-amyloid deposition and neuronal cell death can trigger robust pro-neuroinflammatory activation of microglia, leading to the release of pro-inflammatory cytokines and complement. A vicious cycle of neuropathology, pro-inflammatory glial activation, and excessive neurodegeneration ensues. This pathological cycle affects the BBB integrity and lymphatic drainage, which leads to immune cell infiltration (e.g., T cells) in the brain parenchyma and border zone, immune cell activation, antigen accumulation, and TCR clonal expansion. In this neuroimmune axis model, immunopathogenesis changes can therefore serve as a foundation for designing and developing of disease-modifying therapies for AD. APC, antigen-presenting cells; TCR, T cell receptor

Neuroimmune interactions introduce further heterogeneity in the etiology and neuropathogenic course of AD. In fact, neuroimmune interactions have increasingly emerged as a major focus in neurodegenerative disease research, including AD, Parkinson’s disease (PD), and multiple sclerosis (MS) [131]. Crosstalk between the brain and the peripheral immune system occurs via either the blood-brain barrier (BBB) [132], choroid plexus (CP) [133, 134], or from the meninges [135–137]. Emerging studies have shown that all these brain interfaces undergo structural and/or biological changes during aging and AD and can act as gateways for infiltrating peripheral immune cells into the brain (Fig. 1). Immunoprofiling studies have demonstrated heterogeneity in microglia [55, 138, 139] and peripheral immune cells, including myeloid cells [140], T cells [141, 142], and B cells [137, 143] in AD. Recent studies have also begun to elucidate the role of peripheral immune cells in brain health and neurodegenerative disease. In addition, genes associated with the immune system have increasingly been associated with AD risk in GWAS, e.g., *IGHG1* (Table 1). However, studies to explore the disease-modifying role of peripheral immune cells in human brain are still at a relatively nascent stage. Given the compelling recent evidence for a role of the peripheral immune system in the pathogenesis of AD, future studies are clearly required to fully understand the contribution of peripheral immune cell-related genetics and brain infiltration to brain health and neurodegenerative diseases.

In this review, we highlight immune signaling pathways and genes associated with AD risk, and interactions between innate and adaptive immune cells in AD. We discuss the complexities of AD at the levels of genetics and cellular physiology and their crosstalk with the immune system. We mainly delve into the litany of the role of peripheral immune cells in AD and the mechanisms by which immune cells, such as T cells, influence AD neuropathogenesis (Fig. 1). Lastly, we review the challenges and outlook for developing immune-based therapies for treating and preventing AD.

## Neuroimmune alterations in AD

The healthy brain is an immunologically active organ protected by the resident immune cells and by infiltrating peripheral immune cells [144]. Recent discoveries have highlighted alterations in brain-resident (microglia) and peripheral immune cells (neutrophils, monocytes, T cells, and B cells) as well as crosstalk between innate and adaptive immune cells in the development of AD neuropathology [145–151]. Under homeostatic conditions, it is now clear that the adaptive immune system is present. Though low in levels, adaptive immune cells, including T cells and B cells, can enter the brain meninges, occupy the dura through skull channels—and play crucial roles in brain maintenance, including neuronal function, brain development, and spatial learning [152], mainly due to cytokines released from T cells, including interleukin-4 (IL-4), IL-17, and IFN-γ [153]. It has been shown that a deficiency of IL-4-producing T cells in the meninges [154] or an excess of these cells in choroid plexus during aging [155] negatively impacts the brain, emphasizing the importance of the adaptive immune system contributing to brain function and maintenance during homeostatic conditions. A recent study on the increased clonal expansion and elevated T cell activation in cytotoxic CD8+ T cells in brains of mild cognitive impairment (MCI) and AD highlight the potential disease-modifying roles of T cells in AD [142]. However, it is yet to be determined whether cytotoxic T cells have a pathogenic role in AD patients. Another possibility is that under disease conditions, such as AD, subsequential damage to the BBB due to the pathological changes may lead to gliosis and infiltration of CD8+ T cells from blood and border zones into the brain parenchyma and subsequent T cell receptor (TCR) clonal expansion. However, it is not fully known whether peripheral CD8+ T cells can cross the BBB and infiltrate into the AD brain parenchyma. In addition, reduced levels of circulating IFN-γ-secreting T cells were linked with age-related cognitive decline in mice [156]. Similarly, lower levels of IFN-γ in the plasma of AD patients were associated with progression of cognitive deficiency [157]. While the underlying mechanisms of increased infiltration of adaptive immune cells in neurodegenerative diseases and aging remain to be elucidated, the role of infiltrating T cells on disease progression differs extensively depending on the specific neurodegenerative disease under investigation and their functional programming [153]. In this section, we summarize the current status of research on the role of innate and adaptive immunity and crosstalk between these systems (Fig. 1) in the AD brain.

## Microglial activation in AD

Microglia, the primary innate immune cells of the brain are crucial for local immune defense, clearing debris and toxins, as well as for maintaining brain homeostasis [158]. Microglia are also critical during brain development, for maintenance of neuronal networks, and for repair following injury or infection [159, 160]. Like sentinels, ramified microglia constantly survey their environment for detrimental signals and switch rapidly to an activated state upon sensing signals indicative of damage, infection, and other pathological conditions [161]. With regard to AD neuropathogenesis, microglial cells play a variety of roles. Conventionally, microglia, like macrophages, have been oversimplistically divided into M1 type (pro-inflammatory) and M2 type (neuroprotective) [162, 163]. However, single-cell transcriptomic and detailed proteomic studies have revised this simple dichotomy by revealing many functionally distinct microglial cell populations covering a spectrum of activities in the healthy brain and in the progression of AD [164]. This includes disease-related microglial cells such as disease-associated microglia (DAM) [165], microglia neurodegenerative phenotype (MGnD) [166], morphologically activated microglia (PAM) [167], and a multitude of unnamed subsets [164].

DAM are characterized by substantially low expression of homeostatic genes and upregulated expression of genes involved in neurodegenerative diseases, including AD and amyotrophic lateral sclerosis (ALS) [165, 166, 168]. Interestingly, genes upregulated in DAM include several known AD-related genes such as *APOE* [169], *CTSD* [170], *LPL* [171], *TYROBP* [172], and *TREM2* [13, 14, 62, 173]. Major players in converting homeostatic microglia to MGnD are controlled by the *APOE/TREM2/CD33* pathways [166]. The molecular DAM signature is defined by upregulated expression of genes related to phagocytosis, chemotaxis, and release of cytokines upon neuronal injury and is accompanied by a suppression of homeostatic genes [166, 174]. Furthermore, the PAM population is associated with Aβ and Tau pathology and the progression of cognitive decline [167, 175]. Nonetheless, microglia functional and phenotypic conversion in AD development and progression has yet to be fully understood with regard to distinguishing between protective and detrimental microglial function.

One of the classic pathological features of AD is nebulously referred to as “microglial activation”, which can be observed over the course of AD neuropathogenesis, possibly even a decade before the onset of clinical symptoms when deposition of β-amyloid plaques first takes place [176]. Increased microglial activation and the transition to DAM can either lead to a neuroprotective effect through Aβ clearance in the early and asymptomatic stages or, as AD pathology progresses, to a persistent inflammatory response leading to neurodegeneration [177]. In mice and AD patients, DAM colocalize with β-amyloid deposits. It has also been shown that DAM can even form a barrier to reduce further deposition of plaques [178, 179] and actively participate in the disassembly and digestion of β-amyloid plaques [165]. However, this process of colocalization can also trigger pro-inflammatory microglial activation and release of pro-inflammatory cytokines. The transition into MGnD is driven by an increase of *APOE* followed by an upregulation of *CLEC7A* expression and a further suppression of homeostatic genes [166]. MGnD are induced by phagocytosis of apoptotic neurons and colocalize with neuritic plaques, which are a hallmark of neurodegeneration in AD [166]. Microglia can also exacerbate the propagation of β-amyloid plaques by triggering the *NLRP3* inflammasome and release of apoptosis-associated speck-like protein containing a C-terminal caspase recruitment domain (ASC specks), which quickly bind and cross-seed Aβ peptides extracellularly [180].

Microglia have also been shown to play a role in phagocytosis and propagation, e.g., via exosome secretion of p-Tau [181, 182]. For instance, chronic microglial activation, induced by excessive deposition of β-amyloid or by localized events of neurodegeneration, can lead to both a maladapted inflammatory response and the intraneuronal accumulation of p-Tau [183]. The presence of pathogenic p-Tau aggregates can further exacerbate pro-inflammatory microglial activation, including generation and release of pro-inflammatory cytokines [184, 185]. This can then trigger the activation of neuronal p38 mitogen-activated protein kinase (p38 MAPK), and further stimulation of Tau hyperphosphorylation [186], leading to a vicious cycle of p-Tau formation, neurodegeneration, and pro-inflammatory microglial activation/neuroinflammation.

Recent studies show that the AD-associated gene, *CD33* [10] promotes pro-inflammatory activation of microglia while *TREM2* promotes phagocytosis and clearance of debris, including Aβ [61, 118]. In addition to this opposing modulation of microglia activation by *CD33* and *TREM2*, the microglial cytosolic protein SHIP1, encoded by the AD-associated gene *INPP5D* is linked to TREM2-signaling and is more highly expressed in plaque-associated microglia and during progression of AD. Inhibition of Inpp5d in 5XFAD mice revealed that it also regulates Aβ pathology and is associated with plaque density [187]. TREM2-dependent upregulation of genes related to phagocytosis and lipid metabolism results in modulating the neuroprotective function of DAM and therefore represents promising targets for enhancing beneficial microglial function and reducing neuroinflammatory events [3]. Moreover, increased CSF soluble TREM2 (sTREM2) and reduced AD risk and age-at-onset are associated with common variants in the AD-associated *MS4A* gene cluster [83]. Activating TREM2 or inhibiting CD33 with humanized antibodies are strategies currently underway in AD clinical trials and represent new therapeutic approaches to treating and preventing AD.

In addition to risk gene identification by GWAS, the integration of a multi-omic dataset allows fine mapping of AD risk loci. For instance, examining the population-level variation of gene expression and incorporating chromatin accessibility could verify previously implicated AD risk genes and identify putative AD genes for loci harboring multiple candidate genes (e.g., *MS4A4E* in the *MS4A* gene cluster) [188]. Such approaches could provide support for the microglial PU.1 transcription factor that has previously been associated with increased AD risk [189, 190]. The PU.1 downstream target genes have a predominantly immune function, in particular, the contributions of myeloid-/leukocyte-related processes were strongly highlighted [188]. This resource for human microglia-specific regulation of transcription provides further evidence for the critical role of microglia in AD. The functional phenotypes of microglia and their multidimensional roles in the etiological and neuropathogenic processes underlying AD are still not very well understood. However, identification of critical genes and pathways continues to be driven by a growing sample size in genetic studies, advances in multi-omic data integration, and targeted functional follow-up analyses. Eventually, these studies will help improve our understanding of microglial contribution to AD and pave the way for new avenues in developing better therapeutics.

## CNS interfaces and peripheral immune cell infiltration

Crosstalk between the brain and the peripheral immune system occurs via three possible routes: (i) BBB, which provides an interface between the brain and circulation; emerging studies demonstrate the BBB breakdown and dysfunction in AD [132], (ii) choroid plexus (CP), which provides an interface between the blood and the CSF and acts as a gateway for bone-marrow-derived immune cell entry into the brain [133, 134], and (iii) meninges, an immune-blood-brain interface that allows immune cells to bypass the BBB and enter to the brain through specialized skull bone marrow channels [135, 136]. Changes in these CNS borders with advancing age could etiologically initiate disease pathology or exacerbate neuropathogenesis. CP dysfunction exhibits fibrosis, an increase in type I interferons (IFN) and local neuroinflammation, and impaired CP transportation function reduces Aβ clearance in the AD brain [134, 156, 191, 192]. Evidence for T cell infiltration into the brain parenchyma through meningeal lymphatic vessels suggests a broader role for peripheral immune cells in both healthy and diseased brains [193, 194].

Moreover, recent insights into the functions and communications between the glymphatic system and meningeal lymphatics in CNS disorders have recognized new important players in neurophysiology [195, 196]. Based on the lymphatic system, CSF flows directionally within the brain leading to non-selective clearance of metabolic wastes, including Aβ and Tau [197–200]. Disturbances in glymphatic efflux due to, e.g., sleep disorders or chronically impaired glymphatic system have been associated with neurodegenerative diseases such as AD [195, 201–204]. Reduction in meningeal lymphatic drainage has also been linked with aging-associated cognitive decline and an impaired glymphatic system to recirculate CSF through the brain [193, 205]. On the basis of these findings, an aging-related deficit in C-C chemokine receptor type 7 (CCR7) contributes to a reduction in glymphatic influx, cognition, and increased β-amyloid deposits in the brain of 5XFAD mice [206]. It is out of the scope of this review to delve into the immune cell compartmentalization and their changes with brain aging and neurodegenerative diseases, which has been recently reviewed elsewhere [144]. We, therefore, in the following sections, focused on discussing recent findings on infiltrating peripheral immune cells (Table 2), and their beneficial/detrimental implications in AD-related neurodegeneration.

**Table 2:** Cellular infiltration in Alzheimer’s disease

**Cell type**	**Model**	**Age**	**Disease-modifying role or correlation**	**Possible recruitment signal**	**References**
Neutrophils	Human	Average age is 74.5 ± 10.4 for AD subjects	Unknown	Unknown	Zenaro et al., 2015 [207]
Neutrophils	5XFAD and 3x-Tg mice	4-month-old (5XFAD), 6-month-old (3xTg mice)	Depletion or inhibition of neutrophils trafficking reduced AD-like pathology and improved cognitive function	LFA-1 integrin	Zenaro et al., 2015 [207]
Monocytes	App_swe_/PS1 mice	6–9-month-old	Reduced Aβ burden	Unknown	Malm et al., 2005 [208] Simard et al., 2006 [209]
Monocytes	Tg2576/TGF-β DNR mice	17–18-month-old	Reduced cerebral parenchymal and vascular β-amyloid deposits	TGF-β signaling	Town et al., 2008 [210]
Monocytes	APP/PS1 mice	6–7-month-old	Depletion of monocytes increased β-amyloid deposition	Aβ aggregates on blood vessels	Michaud et al., 2013 [211]
Monocytes	5XFAD mice	10-month-old	PD-1 blockade led to clearance of cerebral β-amyloid plaques and improved cognitive performance	PD-1/IFN-γ pathway	Baruch et al., 2016 [212]
CD8+ T cells	Human	16–81-year-old (Rogers, 1988), 56–96-year-old (Togo, 2002) for AD subjects	Likely have negative consequences for neuronal function and integrity	Unknown	Rogers et al., 1988 [213] Togo et al., 2002 [214]
CD8+ T cells	Human	Average age is 84.1 ± 3.6 for AD subjects	Unknown	Unknown	Merlini et al., 2018 [215]
CD8+ T cells	Human	72–96-year-old for AD subjects	Positive correlation of parenchymal CD8+ T cells with Braak stage	Unknown	Unger et al., 2020 [216]
CD8+ T cells	Human	Average age is 70.74 ± 7.01 for AD subjects	Positive correlation of peripheral T_CM_ and T_EM_ with cognition, and negative correlation of peripheral T_EMRA_ with cognition	Unknown	Gate et al., 2020 [142]
T cells	APP/IFN-γ Tg mice	9-month-old	Clearance of Aβ	IFN-γ cytokine	Monsonego et al., 2006 [217]
CD4+ and CD8+ T cells	ArcAβ mice	12- and 22–24-month-old	No association with β-amyloid deposits	Aβ-induced endothelial cell activation	Ferretti et al., 2016 [218]
CD4+ and CD8+ T cells	APP/PS1 mice	6–7-month-old (Browne, 2013), 10-month-old (McManus, 2017), 18–19-month-old (Unger, 2020), 12–13-month-old (Gate, 2020)	Possible contribution of CD8+ T cells to neuronal dysfunction and modulation of synaptic plasticity	Unknown	Browne et al., 2013 [219] McManus et al., 2017 [220] Unger et al., 2020 [216] Gate et al., 2020 [142]
CD4+ and CD8+ T cells	5XFAD mice	12-month-old	TNF inhibitor treatment reduced CD4+ T cells in the brain, rescued impaired LTP, and decreased β-amyloid plaques	sTNF/TNFR1 signaling	MacPherson et al., 2017 [221] Shukla et al., 2019 [222]
Aβ-specific CD4+ T_H_1 cells	5XFAD mice	9-month-old	ICV-injected T_H_1 cells decreased β-amyloid plaques	Unknown	Mittal et al., 2019 [223]
Aβ-specific CD4+ T_H_1 and T_H_17 cells	APP/PS1 mice and Aβ_42_-induced rats	4–5-month-old (Machhi, 2021), 4-month-old (Zhang, 2013)	Teatment with T_H_1 and T_H_17 cells in APP/PS1 mice accelerated memory impairment and systemic inflammation, increased amyloid burden, activated microglia, and exacerbated neuroinflammation; T_H_17 cells mediated neuroinflammation and neurodegeneration in Aβ_42_-induced rats	Unknown	Machhi et al., 2021 [224] Zhang et al., 2013 [225]
CD4+ T cells	3xTg mice	6–9-month-old	$\alpha$4 blockade reduced Aβ load, Tau hyperphosphorylation and memory decline	$\alpha$4β1 integrin-VCAM-1 signaling	Pietronigro et al., 2019 [226]
T_reg_ cells	5XFAD mice	10-month-old	PD-1 blockade led to clearance of cerebral β-amyloid plaques and improved cognitive performance	PD-1/IFN-γ pathway	Baruch et al., 2016 [227]
T_reg_ cells	APP/PS1 mice	4–7-month-old	Depletion of T_reg_ cells accelerated cognitive deficits and amplification of T_reg_ cells by IL-2 treatment increased plaque-associated microglia, and improved cognitive functions	Unknown	Dansokho et al., 2016 [228]
B cells	3xTg mice	14–16-month-old	Depletion of B cells reduced β-amyloid plaque burden and microglial activation	Unknown	Kim et al., 2021 [229]

*T*_*CM*_ central memory T cells, *T*_*EM*_ effector memory T cells, *T*_*EMRA*_ terminally differentiated memory T cells, *sTNF* soluble tumor necrosis factor, *TNFR1* tumor necrosis factor receptor 1, *TGF-β* transforming growth factor beta, *PD-1* programmed death-1, *IFN-γ* interferon gamma, *LFA-1* lymphocyte function-associated antigen 1, *ICV* intracerebroventricularly, *LTP* long-term potentiation

## BBB breakdown during AD pathogenesis

As mentioned above, one possible communication route between the CNS and periphery is the BBB, which comprises various cell types, including endothelial cells, astrocytes, pericytes, and smooth muscle cells [230]. The BBB protects the central compartment by selective regulation and transportation of neurotoxins and serum factors via specialized tight junctions and transporters [231]. Vascular contributions to dementia and AD are increasingly being elucidated [132, 232–236]. In experimental imaging and pathological and epidemiological studies, dysfunction in the BBB and each of the cellular components of the neurovasculature have been linked to AD. These observations have led to the “AD neurovascular hypothesis,” which suggests that cerebrovascular impairment contributes to and perhaps even initiates AD pathogenesis and cognitive decline. Vascular dysfunction in AD accelerates BBB breakdown [237–242], degeneration of pericytes [237, 240, 243, 244], and reduction of BBB-associated cells that maintain integrity [239, 243, 245–251]. The presence of vascular Aβ pathology, also called cerebral amyloid angiopathy (CAA), predisposes toward neurovascular impairment and, sometimes, stroke [252]. Recent studies have suggested that BBB dysfunction is correlated with human cognitive impairment [239], including the early clinical stages of AD, and is considered as an early biomarker of the disease [132, 235, 253, 254]. Moreover, the identification of BBB impairment as an aging risk factor has been connected to the presence of peripheral immune cells, e.g., T cells, in the brains of the elderly and patients with age-associated neurodegenerative diseases [132, 255–259]. Pathogenic infiltration of peripheral immune cells into the brain parenchyma may lead to exacerbation of AD pathology [146, 149, 256]. Thus, reducing BBB breakdown could serve as a potentially useful therapeutic approach for limiting the infiltration of detrimental peripheral immune cells into the CNS.

## Peripheral innate immunity in AD

Neutrophils are considered the first line of defense in our body during pathological conditions. Infiltrating neutrophils found in the brains of AD patients and transgenic animal models (Table 2) [207, 260] have attracted growing interest in the last few years in MS and AD [261]. In the blood, the neutrophil:lymphocyte ratio has been correlated with cognitive decline in AD [262, 263]. A limited number of studies demonstrated that neutrophils may contribute to the early stages of AD by mediating BBB damage, intravascular adhesion, and invasion of the CNS [207]. Infiltrating neutrophils also induce neurotoxicity by releasing IL-17, a cytotoxic cytokine for neuronal cells and mediating BBB breakdown [264], neutrophils extracellular traps (NETs), and myeloperoxidase (MPO) [207]. Moreover, depletion of infiltrating neutrophils using anti–Ly 6G or anti–Gr-1 antibody in two mouse models of AD (5XFAD and 3xTg mice) has been shown to significantly reduce the amyloid burden and microglial activation and improve performance in the Y-maze spontaneous alternation task and contextual fear-conditioning test [207], suggesting that neutrophils can play an important role in the development of AD.

Although a handful of studies suggest that pro-inflammatory, CNS-infiltrating neutrophils are detrimental in AD, the field would benefit from a deeper understanding of the causal role of neutrophils, their pathological pathways and mechanisms of action in the process of AD-related neuropathogenesis. A recent study identified a unique subset of neutrophils that can promote neuronal survival in the CNS [265]; the salutary effects of this subset of neutrophils could be therapeutically employed in various neurological disorders, including AD. How neutrophils infiltrate the brain and the factors that determine whether they are detrimental or protective are pivotal questions that need to be explored to gain a deeper insight into AD pathogenesis. Moreover, there is a dire need to understand the interactions between infiltrating neutrophils and brain-resident cells, which could offer new avenues for treating and preventing AD.

Another innate immune cell population consists of monocytes, which are found peripherally and less often in the CNS. Circulating monocytes are heterogeneous cells divided into multiple subsets with different surface markers, heterogeneous transcriptional profiles, and different functions. In AD transgenic mouse models (APP/PS1 and 5XFAD), circulating monocytes (CD14^+^/CD16^−^) can infiltrate the brain (Table 2), reduce Aβ burden, and improve cognitive performance [208, 212], suggesting a beneficial disease-modifying role for these cells in AD pathology. The C-C motif chemokine receptor 2 (CCR2) plays a vital role in the recruitment of monocytes into the CNS. CCR2 blockade in transgenic mouse models of AD (App_swe_/PS1 and Tg2576) led to detrimental effects—increased β-amyloid pathology and exacerbated memory deficits [266, 267]. Live two-photon imaging studies have shown that patrolling monocytes crawl onto luminal walls of Aβ^+^ veins, internalize Aβ and circulate back into the bloodstream, suggesting that monocytes play a role in the clearance of vascular Aβ in AD [211]. Monocyte-mediated clearance of Aβ may constitute a unique therapeutic approach for reducing AD pathology using circulating monocytes.

Other studies have shown that blocking transforming growth factor-β (TGF-β) signaling on peripheral macrophages results in substantial infiltration and clearance of cerebral Aβ in the Tg2576 mouse model of AD, suggesting another potential anti-amyloid therapeutic approach [210]. It is important to note that while circulating monocytes can infiltrate the brain and eliminate debris such as cerebral deposits of Aβ, these cells are dramatically less effective in clearing pathology in AD with limited phagocytic ability and phenotypes that have been modulated toward pro-inflammatory conditions compared to monocytes of healthy controls [268]. This is in line with human studies, in which peripheral monocytes reveal reduced capacity for Aβ uptake and phagocytosis [268]. Collectively, these results indicate that the impact of chronic neuroinflammatory diseases such as AD on circulating monocytes is still unclear. Additional studies are needed to determine the direct role of circulating monocytes in AD, which may provide a deeper understanding of the underlying mechanisms of AD pathogenesis and lead to novel therapeutic targets for AD and other neurodegenerative diseases.

## The contribution of adaptive immunity to AD pathogenesis

T lymphocytes are a pivotal part of the adaptive immune system, and cumulative evidence suggests that adaptive immune cells influence the pathophysiology of neurodegenerative diseases such as AD. Post-mortem brains from AD patients and corresponding AD-like animal models reveal that CD4+ and CD8+ T cells infiltrate the AD brain (Table 2) [142, 213–222]. However, their precise role in AD pathogenesis remains unclear. Although the pathogenic role of infiltrating CD4+ T cells has been controversial in different neurological disorders, there are only a handful of studies that have revealed a beneficial role of infiltrating CD4+ T cells into the brain parenchyma that target β-amyloid plaques, promoting Aβ clearance and neuronal repair [269]. The mechanisms underlying CD4+ T cell infiltration and activation in AD brain are unclear. Additional studies are required to identify underlying mechanisms of infiltrating CD4+ T cells in the course of AD and whether their infiltration has a beneficial impact on the disease pathology. Limited observations revealed that $\alpha$4-integrins on the surface of peripheral CD4+ T cells are highly expressed in the 3xTg mouse model versus wild type, and these cells infiltrate near vascular cell adhesion protein 1 (VCAM-1)+ cerebral vessels [226]. Blockade of $\alpha$4β1 integrin-VCAM-1 signaling reduced leukocyte adhesion to cerebral vessels and activated microglial cells, and improved memory in the 3xTg mouse model, suggesting a detrimental role of CD4+ T cells, in contrast to previous studies [226]. The harmful effect of CD4+ T lymphocytes in the pathogenesis of AD was also demonstrated via infiltration of T helper 17 (T_H_17) cells, a subtype of CD4+ T cells into the brain parenchyma, resulting in an increased level of IL-17 and IL-22 cytokines in the CSF, serum, and hippocampus of AD models. Infiltrating T_H_17 cells also lead to neuronal apoptosis [225]. Interestingly, serum levels of IL-17 in AD patients have been shown to be elevated [270, 271], and similar observations of infiltrating T_H_17 cells into the brain and increased levels of IL-17 in the CSF and blood have been reported in MS patients [272–274].

The impact of CD4+ T cells on neurodegeneration varies depending mostly on their subsets. Regulatory T (T_reg_) cells have been associated with diverse neuroinflammatory and neurodegenerative diseases such as AD due to their regulatory characteristics. However, their contribution to AD neuropathogenesis remains controversial. Depletion of T_reg_ cells in the APP/PS1 mouse model reduced recruitment of β-amyloid plaque-associated microglial cells and accelerated cognitive impairment [228]. By contrast, depletion of T_reg_ cells in the 5XFAD mouse model has been linked with clearance of β-amyloid plaques and increased recruitment of immune cells through the CP [227]. In this line and to specifically amplify T_reg_ cell populations, treatment of APP/PS1 AD mice with low-dose peripheral IL-2 administration increased microglia recruitment to β-amyloid plaques and restored memory function [228]. Collectively, these studies suggest that T_reg_ cells play an important role at the early stages of AD in regulating resident microglial cell-mediated clearance of parenchymal deposits of β-amyloid. However, additional studies are necessary to dissect the precise role of T_reg_ cells in AD etiology, their underlying mechanisms and whether therapeutic modulation of T_reg_ cells in AD is beneficial.

Human studies and AD transgenic animal models have shown that infiltration of cytotoxic CD8+ T cells correlates with a worsening disease, suggesting a role for these cells in AD pathogenesis [213, 214, 216]. Blood immune profiling of AD patients and healthy individuals has revealed a higher percentage of activated HLA-DR+ CD8+ T cells and augmented release of pro-inflammatory cytokines [142, 275], suggesting that circulating cytotoxic T cells are activated in the blood of AD patients. Notably, a recent study powerfully demonstrated the clonal expansion of CD8+ T cells in the brains/CSF of MCI and AD patients, indicating CD8+ T cells may impact neurodegeneration and/or cognitive impairment in AD [142]. Collectively, these findings underscore the critical pathogenic roles of infiltrating T cells in AD. However, to date, these studies have not yet provided direct causative evidence for infiltrating T cells playing an etiological or disease-modifying role in AD. This is partially due to a lack of a comprehensive disease model that recapitulates T cell infiltration and interaction with human brain cells with different human genetic backgrounds.

T cells and microglia crosstalk has been shown to help maintain homeostasis and shape neuropathology during chronic neurodegeneration [276]. Several studies have suggested that crosstalk between microglia and infiltrating CD4+ T cells plays a critical role in orchestrating immunoregulatory mechanisms in AD pathogenesis [223, 224]. For example, Aβ-specific CD4+ T helper 1 (T_H_1) cells induce a major histocompatibility complex class II (MHC II)+ population of microglia that abrogate AD-like pathology in the 5XFAD mouse model, likely due to interferon gamma (IFN-$\gamma$) cytokine signaling [223]. In contrast, injecting Aβ-specific CD4+ T_H_1 and T_H_17 T_eff_ cells into the brains of APP/PS1 mice has been shown to exacerbate Aβ burden, microgliosis, neuroinflammation, and cognitive impairment [224]. Given the observation of T_reg_ deficits in AD, this may be partially due to breaking immune tolerance by limiting T_reg_ cells in the circulation and CNS, thus compromising T_reg_ immunosuppressive functions [228]. These studies underscore a critical and complex disease-modifying role for CD4+ T cells in AD pathogenesis. Additionally, few other studies revealed the role of microglia as antigen-presenting cells (APCs) to mediate CD8+ T cell infiltration during viral infection [218, 277, 278], which might be relevant to neurodegenerative diseases, including AD and PD. To assess whether age-related T cell infiltration is due to passive extravasation or promoted by microglia as APCs via MHC II receptors, a study using monkeys found that T cell entry into the brain is correlated with activated microglial cells and cognitive impairment [279]. This can go in the other direction in which infiltrating T cells possibly alter microglia phenotypes, neuroinflammation, and neurodegeneration. Thus, it will be important to investigate the impact of the peripheral immune cells, including infiltrating CD8+ T cells and different subsets of CD4+ T cells, as well as other cells, on microglia and their interaction consequences on neuronal cells during AD pathogenesis [280].

In contrast to T cells, the role of B cells and their involvement in AD has been relatively less explored. Mature B cells have been reported in the brains of AD transgenic mice using single-cell RNA sequencing data [173]. Consistent with this observation, recent evidence reveals infiltration of B cells into the brain parenchyma of 3xTg AD mouse model (Table 2), which results in elevated IgG around β-amyloid plaques, activated microglial cells, and has been linked to heightened progression of AD pathology [229]. Depletion or inactivation of B cells at the early stages of AD pathology in transgenic AD mice has demonstrated beneficial therapeutic impact by restoring TGF-β^+^ microglia, which have enhanced ability to clear Aβ oligomers and slow down the progression of AD. The loss of B cells has been shown to significantly reduce β-amyloid plaque burden and reverse behavioral and memory deficits in the 3xTg AD mouse model [229]. This study suggested that while B cells infiltrating the brain parenchyma can produce what may be beneficial IgG around β-amyloid plaques, they also exacerbate the manifestation of AD-like pathology. Although the exact role of B cells in AD neuropathogenesis is still in its infancy, depletion of a specific subset of infiltrating B cells may offer a unique disease-modifying treatment similar to the use of anti-CD20 antibodies in relapsing-remitting and primary progressive MS [281, 282]. The dominant mode of action of most of these antibodies is through selective immunosuppression of pathogenic immune cells (B cells and a small population of T cells), for instance, by blocking α4β1 integrins to halt infiltration of these immune cells to the CNS [283, 284]. Of note that these immunotherapies are effective in controlling inflammation in MS patients with ongoing inflammation, but they fail to halt the disease progression, and their effects are often short-lived [285]. Decelerating the multifaceted vicious cycle of AD neuropathology will be even more challenging.

## Role of systemic inflammation in AD

Aging has been linked to alterations in the systemic immune system associated with an increased frequency of inflammation and infection [149]. Emerging evidence suggests that manifestations beyond the brain include systemic inflammatory events (e.g., circulating pro-inflammatory cytokines and chemokines or common cold), early-life or long-life exposure to infection agents (e.g., herpes simplex virus and *Chlamydophila pneumonia*), or critical disease (e.g., sepsis) have been associated with an increased risk of developing AD and cognitive decline [286]. Investigating this periphery-brain interaction may provide new insights into the understanding of AD pathogenesis. It could offer great promise for novel therapeutic and diagnostic approaches. Studies have suggested that even a single recent infection can modulate peripheral immune-brain communication and accelerate cognitive decline in AD patients and elderly adults [287–289]. For example, increased serum levels of pro-inflammatory cytokines tumor necrosis factor–α (TNFα) and IL-6 are directly linked with neuropsychiatric features in AD [289], suggesting that such systemic inflammatory events may further promote the disease progression. TNFα and vascular endothelial growth factor (VEGF) in combination with Aβ_1-42_ were also found to reduce viability of neurons in culture [290]. A study performed in the Han Chinese population found increased serum levels of IL-18, IL-23, and IL-17 in AD patients compared to healthy controls [271]. This observation also confirmed in APP/PS1 mice, where it was found that increased level of IL­12/IL­23 subunit p40 correlates with reduced amyloid burden in APP/PS1 mice lacking IL-12 and IL-23 [291]. Furthermore, a significant linear correlation of cognitive performance and CSF p40 values in AD and control subjects were observed [291], indicating that IL­12/IL­23 signaling plays a crucial role in regulating not only the amount of β-amyloid plaques, but also cognitive impairment.

In contrast to the results from previous observations regarding TNFα, IL-6, and IL-12, we have recently shown that increased plasma levels of the pro-inflammatory cytokines, IL-12, and IFN-*γ* have been associated with a healthier cognitive trajectory in normal non-demented elderly, particularly in those with β-amyloid-positive brains [157]. Increased levels of these two cytokines would be expected to ramp up T cell-macrophage interactions leading to enhanced defense against infection. Given our recent findings showing that Aβ is an antimicrobial peptide, this is particularly interesting in view of the antimicrobial protection hypothesis of AD [292], which posits Aβ aggregation and subsequent β-amyloid deposition can be triggered by microbial infection in the brain. Perhaps, by affording enhanced protection from peripheral infection, higher levels of plasma IL-12 and IFN-*γ* may also reduce entry of pathogens into the brain, which would otherwise trigger the antimicrobial response of Aβ to form plaques [157]. Along similar lines, recent studies have also shown that various types of vaccination, from influenza to bacillus Calmette-Guérin (BCG), protect against AD risk [293–298], further suggesting that protection against common infections may help reduce AD neuropathology, along the lines of the antimicrobial protection hypothesis [292].

Recent studies also have attempted to determine whether acute episodes of systemic inflammation influence the risk for AD. In one study, short-term systemic inflammatory attacks were linked with increased serum levels of TNFα cytokine and an enhanced rate of cognitive decline in AD subjects [299]. Another recent study demonstrated that a history of severe infections requiring hospital admission and treatment was associated with an increased long-term risk of vascular dementia and AD [300]. These infections were not limited to CNS infections and covered a variety of hospital-treated viral and bacterial infections, suggesting that exposure to systemic inflammation is sufficient to affect the brain and increase the risk of dementia. These studies collectively underscore the potential roles of infections and systemic inflammatory events in the etiology of dementia and AD. Moreover, they raise the question of whether practices and strategies to improve infection control and general inflammation might mitigate or delay AD risk. There is, of course, a caveat to note that hospitalization per se (and not the infection itself) could also be associated with greater dementia or AD risk [301–303].

## AD therapeutics: challenges and opportunities

Providing millions of people living with a debilitating disease like AD with effective treatment is a monumental challenge that clinicians and scientists have faced since the first description of the disease by Alois Alzheimer in 1906. Moreover, preventing AD is of equal importance as our population ages at a dramatic rate thereby increasing the prevalence of AD. Thus far, efforts to treat the disease have been marginally successful in managing symptoms, but with essentially no impact on modification of disease progression. Because of the heterogeneity of AD and complex nature of the brain, effective drug discovery and development will require well-coordinated investigations of the molecular, cellular, and genetic factors involved in AD neuropathogenesis as well as crosstalk between the brain and peripheral immune system.

**Fig. 2:**
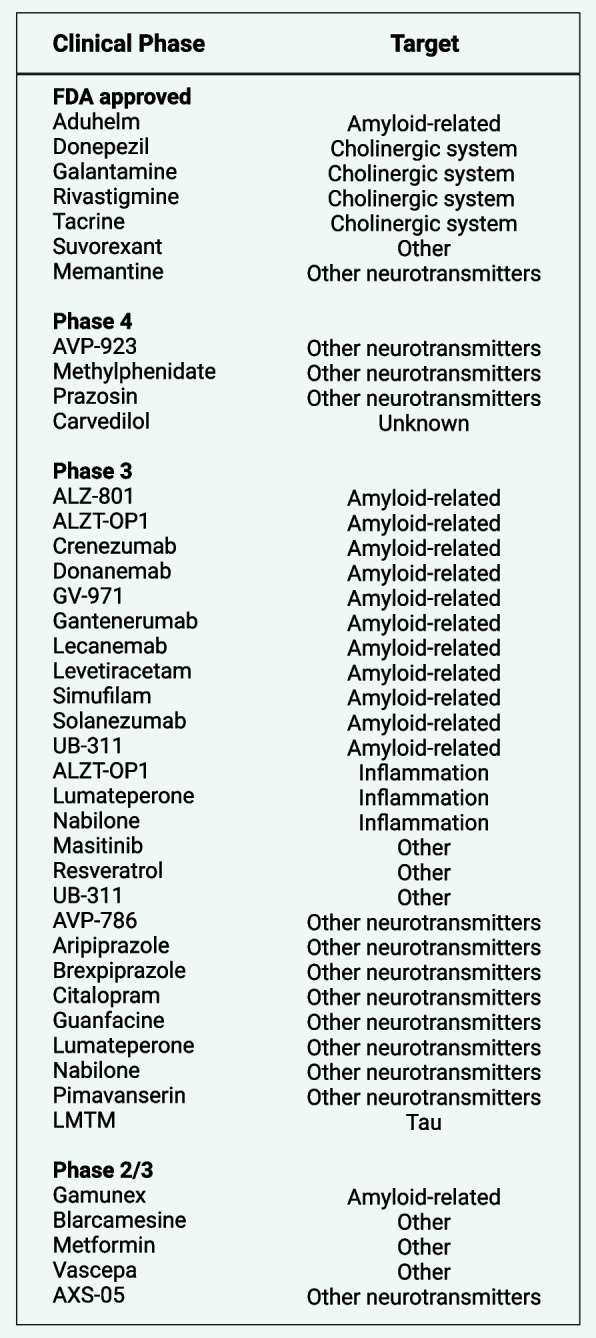
Investigational drugs and proposed targets for the treatment of Alzheimer’s disease and related dementia (ADRD), focusing on those that have been approved or progressed to Phase 2/3 or beyond in U.S. clinical trials. This up-to-date dataset was obtained from alzforum.org, a resource provided by FBRI LLC

The development of potential therapeutics to treat and prevent AD has been enormously challenging over the past decades, leading to no disease-modifying drugs. The investigational drugs and proposed targets of AD that have progressed to Phase 2 or 3 clinical trials in the U.S. are summarized in Fig. 2. Although the recent U.S. Food and Drug Administration (FDA) approval of aducanumab and its clinical impact is highly controversial [304, 305], it is a promising sign that we can target specific pathological hallmarks of AD. Aducanumab is an immunotherapy (Biogen) derived from memory B cells developed initially by the Swiss company, Neurimmune. The search for an Aβ immunotherapy at Neurimmune was based on identifying naturally occurring protective human antibodies targeting Aβ oligomers, originally inspired by the findings of Moir et al. [306]. The controversy regarding FDA approval of aducanumab was initially ignited by the Biogen company as they halted two phase III trials of aducanumab as the interim analysis showed that the drug was unlikely to improve cognition of mild AD patients. However, re-evaluation of the data revealed a subset of people that might have benefited, which lead to submission of aducanumab for FDA approval. This is further fueled by the unusual route of an “accelerated approval” pathway of the FDA, which is reserved for treatments that are “reasonably likely,” to help patients, but not certain.

Several other forms of disease-modifying therapeutics are under development for AD or being clinically tested, e.g., small molecules that target γ-secretase enzyme to reduce Aβ peptide production. γ-secretase modulators selectively decrease the level of Aβ42 over Aβ40 and potentiate formation of nonfibrillar and shorter Aβ peptide species, including Aβ37 and Aβ38 [307]. Since β-amyloid deposition begins one to three decades before symptoms [308], anti-Aβ therapies, e.g., Aβ immunotherapy and γ-secretase modulators [307] would be best used pre-symptomatically as a prophylactic or means of secondary prevention following a positive test for Aβ accumulation in the brain, e.g., by PET or blood test. While most of attempts to improve cognitive symptoms in AD patients by reducing Aβ levels in the brain have largely failed, owing to a number of reasons that have been comprehensively discussed elsewhere [309–311], but new promising results from Biogen and Eisai clinical trial earlier this year bring new hopes to people afflicted with this memory-robbing neurodegenerative disease [312]. This anti-amyloid monoclonal antibody, called lecanemab, showed 27% slower progress in cognitive decline in people with early-stage AD compared to placebo, which might likely be due to its mechanism of action in targeting “protofibrils” strands at earlier stages of the disease before they consolidate into β-amyloid plaques and the length of the trial (i.e., 18 months) that allowed showing a meaningful impact on cognition. Moreover, development of bispecific antibodies by linking them to a BBB transporter moiety may facilitate BBB passing into the brain and improve antibody design, which enables targeting soluble Aβ aggregates with a wide range of sizes in a mouse model [313]. Even though the beneficial effects of Aβ immunotherapy on AD are still uncertain, increasing delivery through the BBB and enhancing antibody binding as well as selectivity to the toxic Aβ aggregates could potentially pave the way to promising therapeutic applications [313].

The identification of AD risk genes by GWAS (Table 1) and whole genome/exome sequencing has progressively expanded our current understanding of AD and emphasized the key role of immune genes involved in AD pathophysiology [3]. New opportunities are emerging for the development of genetic risk scores used to assess the impact of genetic susceptibility factors in risk prediction models. AD and its genetically heterogeneous nature include subtypes that may not homogeneously respond to a specific intervention. Comprehensive risk profiling provides the opportunity to categorize patient subgroups for gene-specific therapeutics, personalized medicine, and translational genomics [31, 314, 315]. The association between Alzheimer’s β-amyloid deposition and sTREM2 [316] points to the notion that one can leverage treatments of microglia-modulating and anti-amyloid therapeutics by targeting, e.g., *TREM2* or *CD33*. Recent studies showed the promising effects of increasing TREM2 in a mouse model using an agonistic antibody design [317]. However, the temporal component is crucial and a beneficial effect of increasing *TREM2* is observed, especially in the early stage of AD development, highlighting the dynamic role of *TREM2* in modulating β-amyloid deposition and neuritic dystrophy in AD pathogenesis [317, 318]. We previously demonstrated the therapeutic potential of targeting *CD33* leveraging an adeno-associated virus vector-based knockdown and observed reduced amyloid accumulation and neuroinflammation in an APP/PS1 mouse model [319].

Additionally, given that the *APOE* ε2 allele is associated with decreased risk of late-onset AD and has been shown to modulate the immune response of microglia, therapeutic strategies aimed at mimicking the protective effects of the *APOE* ε2 allele are now being considered as disease-modifying interventions for AD [320]. A viral-mediated overexpression of *APOE* ε2 in amyloid mouse model brains led to a reduction of the Aβ burden, which might be attributed to an increased Aβ clearance in *APOE* ε2 expressing animals [321]. Gene delivery of *APOE* ε2 may halt or lessen Aβ burden in the brain and subsequent neuritic plaques and inflammatory processes. However, long-term *APOE* ε2 overexpression in human brains should be carefully evaluated and raises concerns since the *APOE* ε2 allele is associated with a higher risk for other diseases like CAA [322] and stroke [320, 323]. Another limiting factor is that in order to be a successful therapy, *APOE* ε2 overexpression would need to be established before the onset of β-amyloid deposition long before the onset of symptoms in patients, which poses its own challenges [324].

The heterogeneity of the disease carries significant implications for drug development, which must be deeply considered in developing effective disease-modifying therapies for AD. A great deal of study is still needed to better understand the clinical and neuropathological heterogeneity in AD and its impact on the development of more effective diagnostics and therapies for treatment and prevention [325]. The complexity of AD heterogeneity suggests that individualized treatments or a combination of multiple targets at different stages of the disease progression from pre-symptomatic to prodromal to clinical manifestation of cognitive impairment will be needed. Another important aspect is the heterogeneity of the immune response, both spatially and temporally [326–328]. The immune system’s plasticity, driven by cellular heterogeneity, allows it to adopt various phenotypes and genotypes in response to internal and external signaling, which could be instrumental to the disease onset, and progression. A combination of environment and genetics shape the heterogenous immune system and response [329]. The environment can play an important role in shaping the composition of the immune cells present in individuals, e.g., by exposure to infection and the microbiota. For instance, the microbial status can be transferred from the mother to the baby during birth and fetal development. In this context, children delivered by cesarean section can have significantly lower levels of CXCL10 and CXCL11 chemokines in their blood [330]. Shortly after birth, diet (first by milk components and then solid food) shapes the microbial community and development of the immune system, including effector and T_reg_ cells, which could have strong impacts later in life [331]. Moreover, genetics is another key factor in determining the level of cytokines produced by the immune cells in response to stimuli.

In addition, given the extreme complexity and heterogeneity of AD, it will be necessary to stratify AD patient groups through deep phenotyping and genotyping along with the application of algorithms that incorporate comprehensive clinical, imaging, biomarker, and pathology data to limit misclassification bias and enable more precise and predictive models for drug discovery and personalized treatment. Successful therapeutics aimed at prevention of AD would require targeting the earliest signs of pathology in the earliest stages of the disease, e.g., early detection of β-amyloid deposition, NFT formation, and neuroinflammation. Recently, substantial advances have been made in efforts to identify the pre-symptomatic stages of AD using CSF and blood-based biomarkers, including Aβ42/40 ratio, p-Tau phosphorylated at threonine-181, 217, or 231, and neurofilament light (NfL) [308]. These early events of abnormal proteostasis and glial activation initiate the disease process pre-symptomatically and drive widespread neuroinflammation, further modified by peripheral immune cell infiltration, which can either ameliorate or exacerbate neuronal cell death leading to dementia. Detecting and targeting peripheral immunity could initiate an exciting new era in the discovery and development of neuroimmune treatments to treat and prevent neurodegenerative disorders, such as AD [332, 333]. Therapeutic strategies focusing on pathogenic or protective peripheral immune cells could enable new immune-based therapeutic opportunities for neurodegenerative diseases, including immunosuppressive drugs that directly target specific populations of brain-infiltrating T cells and other immune cells, such as anti-CD3 antibodies, TNF antagonists, or calcineurin inhibitors [334]. Moreover, mammalian target of rapamycin (mTOR) inhibitors such as rapamycin may help promote beneficial T_reg_ cells while inhibiting detrimental T_H_17 cells in AD [334].

The role of specific T cell subsets in AD pathophysiology remains to be fully elucidated. Other new immune-based therapeutic approaches, including depletion of B cells at the early stages of the disease, have already shown promise in delaying AD progression in animal models [229]. Future studies are now necessary to explore whether therapeutics targeting beneficial peripheral immune cells, e.g., monocytes, T_reg_ cells, or detrimental cells such as T_H_17 cells, by cell-specific immunotherapies or other strategies will be helpful in treating and preventing AD. For instance, IL-17-producing T cells have been shown as crucial players in promoting BBB disruption and disease progression in multiple neurodegenerative diseases, including AD, PD, and MS [42, 264, 335]. Most importantly, neutralization of IL-17 cytokine was shown to prevent cognitive impairments, synaptic dysfunction, and rescue neuroinflammation in AD animal models [42, 336]. These findings indicate that tuning the exacerbated levels of IL-17 cytokine in AD might be an important therapeutic target to prevent its deleterious effect on disease progression. Conversely, it is known from several investigations that depletion of T_reg_ cells in AD mouse models exacerbates disease progression. In this line, treatment of AD mice with low-dose IL-2 cytokine to specifically amplify T_reg_ cell populations rescued cognitive function [337]. Moreover, the recent CNS-specific gene delivery of IL-2 provides a critical and important therapeutic method for an effective IL-2 delivery system in preclinical models [338]. Although presence of T_reg_ cells in the brain parenchyma is mostly beneficial, a higher level of T_reg_ cells in peripheral blood is associated with immune aging and chronic systemic inflammation [339]. This amplifies the complexity of the immune system and sheds light on the importance of not only the immune cells’ function but also their location, which can dictate different outcomes and inform future studies for designing more efficient therapeutics, e.g., amplification of T_reg_ cells in individuals without inducing systemic immune inflammation.

While immune system heterogeneity has long been acknowledged, limitations of conventional experimental models have constrained our ability to systematically dissect the underlying mechanisms and causes. Recent advances in microfluidic technology, single-cell omics, molecular biology, and imaging now enable the profiling and tracking of immune cells at a single-cell resolution [327]. Single-cell RNA sequencing technologies have had a substantial impact and allow for a better understanding of the immune system heterogeneity and immune function [329]. Also, simultaneous readouts using multi-omic profiling, including cell surface proteins, gene expression, and receptor sequences, provided new insights into a highly heterogeneous immune system. Moreover, more physiologically relevant models (mouse and multicellular in vitro) are needed to accurately predict immune responses to drug treatments in preclinical models of disease. These new advancements will undoubtedly refine our understanding of the highly heterogeneous immune cells and expedite the search for better therapies. Along these lines, patterns of immune response change during the (long) course of progression in neurodegenerative diseases. Interventions that might be beneficial in the early stages of disease could be detrimental in the late stages. Another level of complexity to consider is that this temporal sequence might not be only observed longitudinally but also at a single time point in a patient’s brain since pathology possibly affects the brain in a stage-wise fashion leading to the coexistence of early and late stages of the inflammatory response in different brain regions at a given point in time. New knowledge derived from the heterogeneity of the immune system will accelerate the development of novel and effective immune-based treatments that skew the balance between detrimental and reparative effects to beneficial for AD patients.

Additionally, with the high complexity and phenotypic variability, as well as a high rate of failures from clinical trials of AD, precision medicine has the potential to not only improve the success of the clinical trials but also reduce financial costs and sample sizes [340]. In this line, studying the disease systematically by considering sex differences, risk factors, blood-based biomarkers, disease progression, and responses to therapeutic treatments are a few pillars that are critical for the implementation of precision medicine in finding a cure and increasing diagnostic accuracy for AD.

## Concluding remarks

AD pathology begins a decade or more prior to the onset of cognitive decline. However, existing therapeutics targeting pre-symptomatic “initiating” pathologies of abnormal proteostasis—β-amyloid aggregation and deposition and induction of p-Tau by Aβ oligomers—are most often applied at the onset of clinical symptoms when neuroinflammation has already inundated affected brain regions. As such therapeutics targeting AD-related proteinopathy have largely failed to improve cognitive symptoms and would best be applied when β-amyloid plaque and tangle pathology and glial activation first begin, usually a decade or more before symptoms, in a form of prophylaxis or secondary prevention. Human resilient brains, those revealing abundant levels of plaques and/or tangles, in the absence of cognitive deficits at death, have revealed that excessive neurodegeneration leading to clinical dementia requires robust events of neuroinflammation, e.g., microglial activation, pro-inflammatory cytokine release, and reactive astrocytes [341]. With the advent of dozens of AD genes emerging from GWAS that implicate immune cells, e.g., microglia and innate immune mechanisms, novel therapeutics aimed at attenuating neuroinflammation have entered into clinical trials, e.g., targeting *CD33* and *TREM2* [3]. The major question now remaining to be answered is whether therapies aimed at abating neuroinflammation and neurodegeneration owing to neuroinflammation will be more successful at effectively treating the symptoms of AD than those targeting abnormal proteostasis—plaques and tangles—which may be better suited for prevention.

The precise mechanisms by which infiltration of peripheral immune cells such as T cells is mediated during age-associated neurodegenerative diseases remain to be elucidated. Knowledge gained from studies of other neuroinflammatory conditions may be useful in this regard. For example, in patients with MS, T_H_17 cells disrupt the BBB, infiltrate the brain parenchyma, and promote neuroinflammation through IL-17 and IL-22 [264]. Understanding the causative roles (protective or detrimental) of resident immune cells, particularly microglia, and infiltrating peripheral immune cells such as T cells in AD will hopefully guide therapeutic avenues to target the immune system at different stages of the disease from pre-symptomatic onset of pathology to clinical symptoms.

Dissecting the roles of immune cells in AD pathogenesis has been challenging, and much of the work discussed in this review have been conducted using mouse models. This field would benefit from model systems that recapitulate the roles of peripheral immune cells and vascularization, e.g., in vitro three-dimensional models with increased cellular complexity, incorporating peripheral immune cells and vascular elements, single-cell level imaging and interactions, and patient-derived cells. Advances in microfluidics, multicellular human models, and generating iPSC-derived microglia (microglia-like cells) offer a new toolset to dissect immune signaling and complex cell interactions that go awry in neurodegeneration. The elaboration of such models will be essential for designing future therapeutic strategies targeting immune pathways.

Taken together, AD progression is an outcome of a complex interplay of several key players, from dysfunctional neurons to resident immune cells, microglia to the peripheral immune system, and dissecting this entangled and highly heterogenous circuit will take time. The emerging neuroimmune axis of AD emphasizes the need to, someday, additionally classify patients according to their AD-related immunogenetic status (Table 1) together with deep genotyping/phenotyping of innate and adaptive immune cells (Table 2), both inside and outside of the brain, to assess effects on AD risk and pathogenesis and to guide the most effective therapies for treatment and prevention. Ultimately, the successful implementation of multidisciplinary studies among experts with disparate and complementary areas of expertise across neuroscience (including computational neuroscience), genetics, immunology, neurology, and bioengineering will be necessary to engender the paradigm shift needed to successfully develop effective treatments aimed at modifying, halting, or reversing AD neuropathogenesis.

## Data Availability

Not applicable.

## References

[1] Bertram L, Tanzi RE (2019). Alzheimer disease risk genes: 29 and counting. Nat Rev Neurol.

[2] Tanzi RE, Bertram L (2005). Twenty years of the Alzheimer’s disease amyloid hypothesis: a genetic perspective. Cell.

[3] Griciuc A, Tanzi RE (2021). The role of innate immune genes in Alzheimer’s disease. Curr Opin Neurol.

[4] Knopman DS, Amieva H, Petersen RC, Chételat G, Holtzman DM, Hyman BT (2021). Alzheimer disease. Nat Rev Dis Primers..

[5] Veitch DP, Weiner MW, Aisen PS, Beckett LA, Cairns NJ, Green RC (2018). Understanding disease progression and improving Alzheimer’s disease clinical trials: recent highlights from the Alzheimer’s Disease Neuroimaging Initiative. Alzheimer’s Dementia.

[6] Bertram L, Tanzi RE (2019). Alzheimer disease risk genes: 29 and counting. Nat Rev Neurol.

[7] Chhatwal JP, Schultz SA, McDade E, Schultz AP, Liu L, Hanseeuw BJ (2022). Variant-dependent heterogeneity in amyloid β burden in autosomal dominant Alzheimer’s disease: cross-sectional and longitudinal analyses of an observational study. Lancet Neurol.

[8] Dujardin S, Commins C, Lathuiliere A, Beerepoot P, Fernandes AR, Kamath TV (2020). Tau molecular diversity contributes to clinical heterogeneity in Alzheimer’s disease. Nat Med.

[9] Das SR, Lyu X, Duong MT, Xie L, McCollum L, Flores R (2021). Tau-atrophy variability reveals phenotypic heterogeneity in Alzheimer’s disease. Ann Neurol.

[10] Bertram L, Lange C, Mullin K, Parkinson M, Hsiao M, Hogan MF (2008). Genome-wide association analysis reveals putative Alzheimer’s disease susceptibility loci in addition to APOE. Am J Hum Genetics..

[11] Hollingworth P, Harold D, Sims R, Gerrish A, Lambert J-C, Carrasquillo MM (2011). Common variants at ABCA7, MS4A6A/MS4A4E, EPHA1, CD33 and CD2AP are associated with Alzheimer’s disease. Nat Genet.

[12] Naj AC, Jun G, Beecham GW, Wang L-S, Vardarajan BN, Buros J (2011). Common variants at MS4A4/MS4A6E, CD2AP, CD33 and EPHA1 are associated with late-onset Alzheimer’s disease. Nat Genet.

[13] Guerreiro R, Wojtas A, Bras J, Carrasquillo M, Rogaeva E, Majounie E (2013). TREM2 variants in Alzheimer’s disease. New Engl J Medicine..

[14] Jonsson T, Stefansson H, Steinberg S, Jonsdottir I, Jonsson PV, Snaedal J (2013). Variant of TREM2 associated with the risk of Alzheimer’s disease. New Engl J Med.

[15] Johansson JU, Brubaker WD, Javitz H, Bergen AW, Nishita D, Trigunaite A (2018). Peripheral complement interactions with amyloid β peptide in Alzheimer’s disease: polymorphisms, structure, and function of complement receptor 1. Alzheimer’s Dementia..

[16] Rogers J, Li R, Mastroeni D, Grover A, Leonard B, Ahern G (2006). Peripheral clearance of amyloid β peptide by complement C3-dependent adherence to erythrocytes. Neurobiol Aging.

[17] Crehan H, Hardy J, Pocock J (2013). Blockage of CR1 prevents activation of rodent microglia. Neurobiol Dis.

[18] Crehan H, Holton P, Wray S, Pocock J, Guerreiro R, Hardy J (2012). Complement receptor 1 (CR1) and Alzheimer’s disease. Immunobiology.

[19] Efthymiou AG, Goate AM (2017). Late onset Alzheimer’s disease genetics implicates microglial pathways in disease risk. Mol Neurodegener.

[20] Taylor RP, Lindorfer MA, Atkinson JP (2020). Clearance of amyloid-beta with bispecific antibody constructs bound to erythrocytes. Alzheimer’s Dementia Transl Res Clin Interventions..

[21] Ryan J, Fransquet P, Wrigglesworth J, Lacaze P (2018). Phenotypic heterogeneity in dementia: a challenge for epidemiology and biomarker studies. Front Public Heal.

[22] Jansen IE, Savage JE, Watanabe K, Bryois J, Williams DM, Steinberg S (2019). Genome-wide meta-analysis identifies new loci and functional pathways influencing Alzheimer’s disease risk. Nat Genet.

[23] Redondo-García S, Peris-Torres C, Caracuel-Peramos R, Rodríguez-Manzaneque JC (2020). ADAMTS proteases and the tumor immune microenvironment: lessons from substrates and pathologies. Matrix Biology Plus.

[24] Mazzon C, Anselmo A, Soldani C, Cibella J, Ploia C, Moalli F (2012). Agrin is required for survival and function of monocytic cells. Blood.

[25] Lambert J-C, Heath S, Even G, Campion D, Sleegers K, Hiltunen M (2009). Genome-wide association study identifies variants at CLU and CR1 associated with Alzheimer’s disease. Nat Genet.

[26] Borucki DM, Toutonji A, Couch C, Mallah K, Rohrer B, Tomlinson S (2020). Complement-mediated microglial phagocytosis and pathological changes in the development and degeneration of the visual system. Front Immunol.

[27] Agrawal V, Sawhney N, Hickey E, McCarthy JV (2016). Loss of Presenilin 2 function is associated with defective LPS-mediated innate immune responsiveness. Mol Neurobiol.

[28] Nam H, Lee Y, Kim B, Lee J-W, Hwang S, An H-K (2022). Presenilin 2 N141I mutation induces hyperactive immune response through the epigenetic repression of REV-ERBα. Nat Commun.

[29] Fung S, Smith CL, Prater KE, Case A, Green K, Osnis L (2020). Early-onset familial Alzheimer disease variant PSEN2 N141I heterozygosity is associated with altered microglia phenotype. J Alzheimer’s Dis..

[30] Mendez MF (2017). Early-onset Alzheimer disease. Neurol Clin.

[31] Bellenguez C, Küçükali F, Jansen IE, Kleineidam L, Moreno-Grau S, Amin N, et al. New insights into the genetic etiology of Alzheimer’s disease and related dementias. Nat Genet. 2022;54(4):412–36. 10.1038/s41588-022-01024-z PMC9005347 35379992

[32] Herda S, Raczkowski F, Mittrücker H-W, Willimsky G, Gerlach K, Kühl AA (2012). The sorting receptor sortilin exhibits a dual function in exocytic trafficking of Interferon-γ and Granzyme A in T cells. Immunity.

[33] Mortensen MB, Kjolby M, Gunnersen S, Larsen JV, Palmfeldt J, Falk E (2014). Targeting sortilin in immune cells reduces proinflammatory cytokines and atherosclerosis. J Clin Invest.

[34] Lambrecht BN, Vanderkerken M, Hammad H (2018). The emerging role of ADAM metalloproteinases in immunity. Nat Rev Immunol.

[35] Seshadri S, Fitzpatrick AL, Ikram MA, DeStefano AL, Gudnason V, Boada M (2010). Genome-wide analysis of genetic loci associated with Alzheimer disease. JAMA.

[36] Sudwarts A, Ramesha S, Gao T, Ponnusamy M, Wang S, Hansen M (2022). BIN1 is a key regulator of proinflammatory and neurodegeneration-related activation in microglia. Mol Neurodegener.

[37] Nordhoff C, Hillesheim A, Walter BM, Haasbach E, Planz O, Ehrhardt C (2012). The adaptor protein FHL2 enhances the cellular innate immune response to influenza A virus infection. Cell Microbiol.

[38] Wixler V (2019). The role of FHL2 in wound healing and inflammation. Faseb J.

[39] (EADI) EADI, (GERAD) G and ER in AD, (ADGC) ADGC, (CHARGE) C for H and AR in GE, Lambert J-C, Ibrahim-Verbaas CA, et al. Meta-analysis of 74,046 individuals identifies 11 new susceptibility loci for Alzheimer’s disease. Nat Genet. 2013;45:1452–8. 10.1038/ng.2802 PMC3896259 24162737

[40] Itakura J, Sato M, Ito T, Mino M, Fushimi S, Takahashi S (2017). Spred2-deficiecy protects mice from polymicrobial septic peritonitis by enhancing inflammation and bacterial clearance. Sci Rep-uk..

[41] Ishikawa E, Kosako H, Yasuda T, Ohmuraya M, Araki K, Kurosaki T (2016). Protein kinase D regulates positive selection of CD4+ thymocytes through phosphorylation of SHP-1. Nat Commun.

[42] Brigas HC, Ribeiro M, Coelho JE, Gomes R, Gomez-Murcia V, Carvalho K (2021). IL-17 triggers the onset of cognitive and synaptic deficits in early stages of Alzheimer’s disease. Cell Rep.

[43] Girondel C, Meloche S (2021). Interleukin-17 receptor D in physiology, inflammation and cancer. Frontiers Oncol.

[44] Schulte-Schrepping J, Reusch N, Paclik D, Baßler K, Schlickeiser S, Zhang B (2020). Severe COVID-19 is marked by a dysregulated myeloid cell compartment. Cell.

[45] Utting O, Sedgmen BJ, Watts TH, Shi X, Rottapel R, Iulianella A (2004). Immune functions in mice lacking Clnk, an SLP-76-related adaptor expressed in a subset of immune cells. Mol Cell Biol.

[46] Gu Y, Chae H-D, Siefring JE, Jasti AC, Hildeman DA, Williams DA (2006). RhoH GTPase recruits and activates Zap70 required for T cell receptor signaling and thymocyte development. Nat Immunol.

[47] Guo H, Zhang J, Zhang X, Wang Y, Yu H, Yin X (2015). SCARB2/LIMP-2 regulates IFN production of plasmacytoid dendritic cells by mediating endosomal translocation of TLR9 and nuclear translocation of IRF7. J Immunol.

[48] Heybrock S, Kanerva K, Meng Y, Ing C, Liang A, Xiong Z-J (2019). Lysosomal integral membrane protein-2 (LIMP-2/SCARB2) is involved in lysosomal cholesterol export. Nat Commun.

[49] Mastrogiovanni M, Vargas P, Rose T, Cuche C, Esposito E, Juzans M (2022). The tumor suppressor adenomatous polyposis coli regulates T lymphocyte migration. Sci Adv.

[50] Zhai Y, Celis-Gutierrez J, Voisinne G, Mori D, Girard L, Burlet-Schiltz O (2021). Opposing regulatory functions of the TIM3 (HAVCR2) signalosome in primary effector T cells as revealed by quantitative interactomics. Cell Mol Immunol.

[51] Wightman DP, Jansen IE, Savage JE, Shadrin AA, Bahrami S, Holland D (2021). A genome-wide association study with 1,126,563 individuals identifies new risk loci for Alzheimer’s disease. Nat Genet.

[52] Macdonald F, Loosdregt J van, Zaiss DMW. T cell derived HB-EGF prevents Th17 cell differentiation in an autocrine way. Biorxiv. 2021. 10.1101/2021.02.09.430418.

[53] Sao T, Yoshino Y, Yamazaki K, Ozaki Y, Mori Y, Ochi S (2018). MEF2C mRNA expression and cognitive function in Japanese patients with Alzheimer&apos;s disease. Psychiat Clin Neuros.

[54] Higashiyama S, Abraham JA, Miller J, Fiddes JC, Klagsbrun M (1991). A heparin-binding growth factor secreted by macrophage-like cells that is related to EGF. Science.

[55] Srinivasan K, Friedman BA, Etxeberria A, Huntley MA, van der Brug MP, Foreman O (2020). Alzheimer’s patient microglia exhibit enhanced aging and unique transcriptional activation. Cell Rep.

[56] G’Sell RT, Gaffney PM, Powell DW (2015). Review: A20-binding inhibitor of NF-κB activation 1 is a physiologic inhibitor of NF-κB: a molecular switch for inflammation and autoimmunity. Arthritis Rheumatol.

[57] Gurevich I, Zhang C, Francis N, Aneskievich BJ (2011). TNIP1, a retinoic acid receptor corepressor and A20-binding inhibitor of NF-κB, distributes to both nuclear and cytoplasmic locations. J Histochem Cytochem.

[58] Raju S, Kometani K, Kurosaki T, Shaw AS, Egawa T (2018). The adaptor molecule CD2AP in CD4 T cells modulates differentiation of follicular helper T cells during chronic LCMV infection. Plos Pathog.

[59] Tao Q-Q, Chen Y-C, Wu Z-Y (2019). The role of CD2AP in the pathogenesis of Alzheimer’s disease. Aging Dis.

[60] Lu R-C, Yang W, Tan L, Sun F-R, Tan M-S, Zhang W (2017). Association of HLA-DRB1 polymorphism with Alzheimer’s disease: a replication and meta-analysis. Oncotarget.

[61] Griciuc A, Patel S, Federico AN, Choi SH, Innes BJ, Oram MK (2019). TREM2 acts downstream of CD33 in modulating microglial pathology in Alzheimer’s disease. Neuron.

[62] Kunkle BW, Grenier-Boley B, Consortium ADG, Initiative TEAD, Consortium C for H and AR in GE, Consortium G and ER in ADDGP and ER for AD (2019). Genetic meta-analysis of diagnosed Alzheimer’s disease identifies new risk loci and implicates Aβ, tau, immunity and lipid processing. Nat Genet.

[63] Bis JC, Jian X, Kunkle BW, Chen Y, Hamilton-Nelson KL, Bush WS (2020). Whole exome sequencing study identifies novel rare and common Alzheimer’s-associated variants involved in immune response and transcriptional regulation. Mol Psychiatr.

[64] Sims R, van der Lee SJ, Naj AC, Bellenguez C, Badarinarayan N, Jakobsdottir J (2017). Rare coding variants in PLCG2, ABI3, and TREM2 implicate microglial-mediated innate immunity in Alzheimer’s disease. Nat Genet.

[65] Ivanov AI, Romanovsky AA (2006). Putative dual role of ephrin-Eph receptor interactions in inflammation. IUBMB Life.

[66] Chen F, Liu Z, Peng W, Gao Z, Ouyang H, Yan T (2018). Activation of EphA4 induced by EphrinA1 exacerbates disruption of the blood-brain barrier following cerebral ischemia-reperfusion via the Rho/ROCK signaling pathway. Exp Ther Med.

[67] Hoshino A, Boutboul D, Zhang Y, Kuehn HS, Hadjadj J, Özdemir N (2022). Gain-of-function IKZF1 variants in humans cause immune dysregulation associated with abnormal T/B cell late differentiation. Sci Immunol.

[68] Agostini S, Costa AS, Mancuso R, Guerini FR, Nemni R, Clerici M (2019). The PILRA G78R variant correlates with higher HSV-1-specific IgG titers in Alzheimer’s disease. Cell Mol Neurobiol.

[69] Zehner M, Marschall AL, Bos E, Schloetel J-G, Kreer C, Fehrenschild D (2015). The Translocon protein Sec61 mediates antigen transport from endosomes in the cytosol for cross-presentation to CD8+ T cells. Immunity.

[70] Rhinn H, Abeliovich A (2017). Differential aging analysis in human cerebral cortex identifies variants in TMEM106B and GRN that regulate aging phenotypes. Cell Syst.

[71] Tschopp J, Chonn A, Hertig S, French LE (1950). Clusterin, the human apolipoprotein and complement inhibitor, binds to complement C7, C8 beta, and the b domain of C9. J Immunol Baltim Md.

[72] Zhao Z, Nelson AR, Betsholtz C, Zlokovic BV (2015). Establishment and dysfunction of the blood-brain barrier. Cell.

[73] Ma K, Chen X, Liu W, Chen S, Yang C, Yang J (2022). CTSB is a negative prognostic biomarker and therapeutic target associated with immune cells infiltration and immunosuppression in gliomas. Sci Rep-uk..

[74] Ha S-D, Martins A, Khazaie K, Han J, Chan BMC, Kim SO (2008). Cathepsin B is involved in the trafficking of TNF-α-containing vesicles to the plasma membrane in macrophages. J Immunol.

[75] Okigaki M, Davis C, Falasca M, Harroch S, Felsenfeld DP, Sheetz MP (2003). Pyk2 regulates multiple signaling events crucial for macrophage morphology and migration. Proc National Acad Sci.

[76] Asanomi Y, Shigemizu D, Miyashita A, Mitsumori R, Mori T, Hara N (2019). A rare functional variant of SHARPIN attenuates the inflammatory response and associates with increased risk of late-onset Alzheimer’s disease. Mol Med.

[77] Zamanian-Daryoush M, Lindner DJ, DiDonato JA, Wagner M, Buffa J, Rayman P (2017). Myeloid-specific genetic ablation of ATP-binding cassette transporter ABCA1 is protective against cancer. Oncotarget.

[78] Westerterp M, Gautier EL, Ganda A, Molusky MM, Wang W, Fotakis P (2017). Cholesterol accumulation in dendritic cells links the inflammasome to acquired immunity. Cell Metab.

[79] Fu C, Turck CW, Kurosaki T, Chan AC (1998). BLNK a central linker protein in B cell activation. Immunity.

[80] Han Y, Liu X, Shi B, Xiao R, Gou M, Wang H (2016). Identification and characterisation of the immune response properties of Lampetra japonica BLNK. Sci Rep-uk..

[81] Zhao Y, Niu L-T, Hu L-J, Lv M (2022). Comprehensive analysis of ECHDC3 as a potential biomarker and therapeutic target for acute myeloid leukemia: bioinformatic analysis and experimental verification. Frontiers Oncol.

[82] Orinska Z, Hagemann PM, Halova I, Draber P (2020). Tetraspanins in the regulation of mast cell function. Med Microbiol Immun.

[83] Deming Y, Filipello F, Cignarella F, Cantoni C, Hsu S, Mikesell R (2019). The MS4A gene cluster is a key modulator of soluble TREM2 and Alzheimer’s disease risk. Sci Transl Med.

[84] Kuek LE, Leffler M, Mackay GA, Hulett MD (2016). The MS4A family: counting past 1, 2 and 3. Immunol Cell Biol.

[85] Harold D, Abraham R, Hollingworth P, Sims R, Gerrish A, Hamshere ML (2009). Genome-wide association study identifies variants at CLU and PICALM associated with Alzheimer’s disease. Nat Genet.

[86] Talbot H, Saada S, Naves T, Gallet P-F, Fauchais A-L, Jauberteau M-O (2019). Regulatory roles of sortilin and SorLA in immune-related processes. Front Pharmacol.

[87] Knupp A, Mishra S, Martinez R, Braggin JE, Szabo M, Kinoshita C (2020). Depletion of the AD risk gene SORL1 selectively impairs neuronal endosomal traffic independent of amyloidogenic APP processing. Cell Rep.

[88] Jones RE, Andrews R, Holmans P, Hill M, Taylor PR (2021). Modest changes in Spi1 dosage reveal the potential for altered microglial function as seen in Alzheimer’s disease. Sci Rep-uk..

[89] He T, Yang D, Li X-Q, Jiang M, Islam MS, Chen S (2020). Inhibition of two-pore channels in antigen-presenting cells promotes the expansion of TNFR2-expressing CD4+Foxp3+ regulatory T cells. Sci Adv.

[90] Su X, Liu N, Wu W, Zhu Z, Xu Y, He F (2021). Comprehensive analysis of prognostic value and immune infiltration of kindlin family members in non-small cell lung cancer. BMC Med Genomics.

[91] Lekhraj R, Lalezari S, Aguilan JT, Qin J, Sidoli S, Mowrey W (2022). Altered abundances of human immunoglobulin M and immunoglobulin G subclasses in Alzheimer’s disease frontal cortex. Sci Rep-uk.

[92] Lee J, Chan SL, Mattson MP (2002). Adverse effect of a presenilin-1 mutation in microglia results in enhanced nitric oxide and inflammatory cytokine responses to immune challenge in the brain. Neuromol Med.

[93] Faraco J, Lin L, Kornum BR, Kenny EE, Trynka G, Einen M (2013). ImmunoChip study implicates antigen presentation to T cells in narcolepsy. Plos Genet.

[94] Conus S, Simon H (2010). Cathepsins and their involvement in immune responses. Swiss Med Wkly.

[95] Zavašnik-Bergant V, Schweiger A, Bevec T, Golouh R, Turk V, Kos J (2004). Inhibitory p41 isoform of invariant chain and its potential target enzymes cathepsins L and H in distinct populations of macrophages in human lymph nodes. Immunology.

[96] Li X, Wu K, Edman M, Schenke-Layland K, MacVeigh-Aloni M, Janga SR (2010). Increased expression of cathepsins and obesity-induced proinflammatory cytokines in lacrimal glands of male NOD mouse. Investigative Opthalmology Vis Sci.

[97] Bilbao D, Luciani L, Johannesson B, Piszczek A, Rosenthal N (2014). Insulin-like growth factor-1 stimulates regulatory T cells and suppresses autoimmune disease. Embo Mol Med.

[98] Andersson KME, Wasén C, Juzokaite L, Leifsdottir L, Erlandsson MC, Silfverswärd ST (2018). Inflammation in the hippocampus affects IGF1 receptor signaling and contributes to neurological sequelae in rheumatoid arthritis. Proc National Acad Sci..

[99] Oh SK, Kim D, Kim K, Boo K, Yu YS, Kim IS (2019). RORα is crucial for attenuated inflammatory response to maintain intestinal homeostasis. Proc National Acad Sci..

[100] Lo BC, Gold MJ, Hughes MR, Antignano F, Valdez Y, Zaph C (2016). The orphan nuclear receptor RORα and group 3 innate lymphoid cells drive fibrosis in a mouse model of Crohn’s disease. Sci Immunol.

[101] Chi X, Jin W, Bai X, Zhao X, Shao J, Li J (2021). RORα is critical for mTORC1 activity in T cell-mediated colitis. Cell Rep.

[102] Wang R, Campbell S, Amir M, Mosure SA, Bassette MA, Eliason A (2021). Genetic and pharmacological inhibition of the nuclear receptor RORα regulates TH17 driven inflammatory disorders. Nat Commun.

[103] Fluhrer R, Grammer G, Israel L, Condron MM, Haffner C, Friedmann E (2006). A γ-secretase-like intramembrane cleavage of TNFα by the GxGD aspartyl protease SPPL2b. Nat Cell Biol.

[104] Foucher ED, Blanchard S, Preisser L, Garo E, Ifrah N, Guardiola P (2013). IL-34 induces the differentiation of human monocytes into immunosuppressive macrophages. Antagonistic Effects of GM-CSF and IFNγ. Plos One.

[105] Lin H, Lee E, Hestir K, Leo C, Huang M, Bosch E (2008). Discovery of a cytokine and its receptor by functional screening of the extracellular proteome. Science.

[106] Huai W, Liu X, Wang C, Zhang Y, Chen X, Chen X (2019). KAT8 selectively inhibits antiviral immunity by acetylating IRF3. J Exp Med.

[107] Gabryšová L, Alvarez-Martinez M, Luisier R, Cox LS, Sodenkamp J, Hosking C (2018). c-Maf controls immune responses by regulating disease-specific gene networks and repressing IL-2 in CD4+ T cells. Nat Immunol.

[108] Magno L, Bunney TD, Mead E, Svensson F, Bictash MN (2021). TREM2/PLCγ2 signalling in immune cells: function, structural insight, and potential therapeutic modulation. Mol Neurodegener.

[109] Harder L, Eschenburg G, Zech A, Kriebitzsch N, Otto B, Streichert T (2013). Aberrant ZNF423 impedes B cell differentiation and is linked to adverse outcome of ETV6-RUNX1 negative B precursor acute lymphoblastic leukemia. J Exp Medicine..

[110] Satoh J, Kino Y, Yanaizu M, Tosaki Y, Sakai K, Ishida T (2017). Microglia express ABI3 in the brains of Alzheimer’s disease and Nasu-Hakola disease. Intractable Rare Dis Res..

[111] Bernstein KE, Khan Z, Giani JF, Cao D-Y, Bernstein EA, Shen XZ (2018). Angiotensin-converting enzyme in innate and adaptive immunity. Nat Rev Nephrol.

[112] Draber P, Vonkova I, Stepanek O, Hrdinka M, Kucova M, Skopcova T (2011). SCIMP, a transmembrane adaptor protein involved in major histocompatibility complex class II signaling. Mol Cell Biol.

[113] Bhatt S, Hillmer AT, Girgenti MJ, Rusowicz A, Kapinos M, Nabulsi N (2020). PTSD is associated with neuroimmune suppression: evidence from PET imaging and postmortem transcriptomic studies. Nat Commun.

[114] Kim WS, Guillemin GJ, Glaros EN, Lim CK, Garner B (2006). Quantitation of ATP-binding cassette subfamily-A transporter gene expression in primary human brain cells. NeuroReport.

[115] Steinberg S, Stefansson H, Jonsson T, Johannsdottir H, Ingason A, Helgason H (2015). Loss-of-function variants in ABCA7 confer risk of Alzheimer’s disease. Nat Genet.

[116] Saunders AM, Schmader K, Breitner JC, Benson MD, Brown WT, Goldfarb L (1993). Apolipoprotein E epsilon 4 allele distributions in late-onset Alzheimer’s disease and in other amyloid-forming diseases. Lancet Lond Engl..

[117] Bonacina F, Coe D, Wang G, Longhi MP, Baragetti A, Moregola A (2018). Myeloid apolipoprotein E controls dendritic cell antigen presentation and T cell activation. Nat Commun.

[118] Griciuc A, Serrano-Pozo A, Parrado A, Lesinski A, Asselin C, Mullin K (2013). Alzheimer’s disease risk gene CD33 inhibits microglial uptake of amyloid beta. Neuron.

[119] Crocker PR, Paulson JC, Varki A (2007). Siglecs and their roles in the immune system. Nat Rev Immunol.

[120] Deng M, Chen H, Liu X, Huang R, He Y, Yoo B (2021). Leukocyte immunoglobulin-like receptor subfamily B (LILRB): therapeutic targets in cancer. Antib Ther.

[121] Boisson B, Laplantine E, Prando C, Giliani S, Israelsson E, Xu Z (2012). Immunodeficiency, autoinflammation and amylopectinosis in humans with inherited HOIL-1 and LUBAC deficiency. Nat Immunol.

[122] Esnault S, Kelly EA, Schwantes EA, Liu LY, DeLain LP, Hauer JA (2013). Identification of genes expressed by human airway eosinophils after an in vivo allergen challenge. PLoS ONE.

[123] Rodríguez-Baena FJ, Redondo-García S, Peris-Torres C, Martino-Echarri E, Fernández-Rodríguez R, Plaza-Calonge M del C (2018). ADAMTS1 protease is required for a balanced immune cell repertoire and tumour inflammatory response. Sci Rep-uk.

[124] Kumar DKV, Choi SH, Washicosky KJ, Eimer WA, Tucker S, Ghofrani J (2016). Amyloid-β peptide protects against microbial infection in mouse and worm models of Alzheimer’s disease. Sci Transl Med.

[125] Eimer WA, Kumar DKV, Shanmugam NKN, Rodriguez AS, Mitchell T, Washicosky KJ (2018). Alzheimer’s disease-associated β-amyloid is rapidly seeded by Herpesviridae to protect against brain infection. Neuron.

[126] Jonsson T, Atwal JK, Steinberg S, Snaedal J, Jonsson PV, Bjornsson S (2012). A mutation in APP protects against Alzheimer’s disease and age-related cognitive decline. Nature.

[127] Ruiz A, Heilmann S, Becker T, Hernández I, Wagner H, IGAP (2014). Follow-up of loci from the International Genomics of Alzheimer’s Disease Project identifies TRIP4 as a novel susceptibility gene. Transl Psychiat.

[128] Baker E, Sims R, Leonenko G, Frizzati A, Harwood JC, Grozeva D (2019). Gene-based analysis in HRC imputed genome wide association data identifies three novel genes for Alzheimer’s disease. PLoS ONE.

[129] Sims R, Hill M, Williams J (2020). The multiplex model of the genetics of Alzheimer’s disease. Nat Neurosci.

[130] Marioni RE, Harris SE, Zhang Q, McRae AF, Hagenaars SP, Hill WD (2018). GWAS on family history of Alzheimer’s disease. Transl Psychiat.

[131] Scheiblich H, Trombly M, Ramirez A, Heneka MT (2020). Neuroimmune connections in aging and neurodegenerative diseases. Trends Immunol.

[132] Sweeney MD, Sagare AP, Zlokovic BV (2018). Blood–brain barrier breakdown in Alzheimer disease and other neurodegenerative disorders. Nat Rev Neurol.

[133] Shechter R, Miller O, Yovel G, Rosenzweig N, London A, Ruckh J (2013). Recruitment of beneficial M2 macrophages to injured spinal cord is orchestrated by remote brain choroid plexus. Immunity.

[134] Dani N, Herbst RH, McCabe C, Green GS, Kaiser K, Head JP (2021). A cellular and spatial map of the choroid plexus across brain ventricles and ages. Cell.

[135] Herisson F, Frodermann V, Courties G, Rohde D, Sun Y, Vandoorne K (2018). Direct vascular channels connect skull bone marrow and the brain surface enabling myeloid cell migration. Nat Neurosci.

[136] Cugurra A, Mamuladze T, Rustenhoven J, Dykstra T, Beroshvili G, Greenberg ZJ (2021). Skull and vertebral bone marrow are myeloid cell reservoirs for the meninges and CNS parenchyma. Science.

[137] Brioschi S, Wang W-L, Peng V, Wang M, Shchukina I, Greenberg ZJ (2021). Heterogeneity of meningeal B cells reveals a lymphopoietic niche at the CNS borders. Science.

[138] Böttcher C, Schlickeiser S, Sneeboer MAM, Kunkel D, Knop A, Paza E (2018). Human microglia regional heterogeneity and phenotypes determined by multiplexed single-cell mass cytometry. Nat Neurosci.

[139] Olah M, Menon V, Habib N, Taga MF, Ma Y, Yung CJ (2020). Single cell RNA sequencing of human microglia uncovers a subset associated with Alzheimer’s disease. Nat Commun.

[140] Jordao MJC, Sankowski R, Brendecke SM, Sagar, Locatelli G, Tai YH (2019). Single-cell profiling identifies myeloid cell subsets with distinct fates during neuroinflammation. Science.

[141] Schmiedel BJ, Gonzalez-Colin C, Fajardo V, Rocha J, Madrigal A, Ramírez-Suástegui C (2022). Single-cell eQTL analysis of activated T cell subsets reveals activation and cell type–dependent effects of disease-risk variants. Sci Immunol.

[142] Gate D, Saligrama N, Leventhal O, Yang AC, Unger MS, Middeldorp J (2020). Clonally expanded CD8 T cells patrol the cerebrospinal fluid in Alzheimer’s disease. Nature.

[143] Phongpreecha T, Fernandez R, Mrdjen D, Culos A, Gajera CR, Wawro AM (2020). Single-cell peripheral immunoprofiling of Alzheimer’s and Parkinson’s diseases. Sci Adv.

[144] Croese T, Castellani G, Schwartz M (2021). Immune cell compartmentalization for brain surveillance and protection. Nat Immunol.

[145] Morris G, Berk M, Maes M, Puri BK (2019). Could Alzheimer’s disease originate in the periphery and if so how so?. Mol Neurobiol.

[146] Stephenson J, Nutma E, Valk P, Amor S (2018). Inflammation in CNS neurodegenerative diseases. Immunology..

[147] Webers A, Heneka MT, Gleeson PA (2020). The role of innate immune responses and neuroinflammation in amyloid accumulation and progression of Alzheimer’s disease. Immunol Cell Biol.

[148] Page AL, Dupuis G, Frost EH, Larbi A, Pawelec G, Witkowski JM (2018). Role of the peripheral innate immune system in the development of Alzheimer’s disease. Exp Gerontol.

[149] Bettcher BM, Tansey MG, Dorothée G, Heneka MT (2021). Peripheral and central immune system crosstalk in Alzheimer disease — a research prospectus. Nat Rev Neurol.

[150] Kinney JW, Bemiller SM, Murtishaw AS, Leisgang AM, Salazar AM, Lamb BT (2018). Inflammation as a central mechanism in Alzheimer’s disease. Alzheimer’s Dementia Transl Res Clin Interventions..

[151] Cao W, Zheng H (2018). Peripheral immune system in aging and Alzheimer’s disease. Mol Neurodegener.

[152] Salvador AF, de Lima KA, Kipnis J (2021). Neuromodulation by the immune system: a focus on cytokines. Nat Rev Immunol.

[153] Schwartz M, Abellanas MA, Tsitsou-Kampeli A, Suzzi S (2022). The brain-immune ecosystem: implications for immunotherapy in defeating neurodegenerative diseases. Neuron.

[154] Derecki NC, Cardani AN, Yang CH, Quinnies KM, Crihfield A, Lynch KR (2010). Regulation of learning and memory by meningeal immunity: a key role for IL-4. J Exp Med.

[155] Baruch K, Ron-Harel N, Gal H, Deczkowska A, Shifrut E, Ndifon W (2013). CNS-specific immunity at the choroid plexus shifts toward destructive Th2 inflammation in brain aging. Proc National Acad Sci.

[156] Baruch K, Deczkowska A, David E, Castellano JM, Miller O, Kertser A (2014). Aging-induced type I interferon response at the choroid plexus negatively affects brain function. Science.

[157] Yang H, Zhang C, Carlyle BC, Zhen SY, Trombetta BA, Schultz AP (2022). Plasma IL-12/IFN-γ axis predicts cognitive trajectories in cognitively unimpaired older adults. Alzheimer’s Dementia.

[158] Madore C, Yin Z, Leibowitz J, Butovsky O (2020). Microglia, lifestyle stress, and neurodegeneration. Immunity.

[159] Ji K, Akgul G, Wollmuth LP, Tsirka SE (2013). Microglia actively regulate the number of functional synapses. PLoS ONE.

[160] Bartels T, Schepper SD, Hong S (2020). Microglia modulate neurodegeneration in Alzheimer’s and Parkinson’s diseases. Science.

[161] Bohlen CJ, Friedman BA, Dejanovic B, Sheng M (2019). Microglia in brain development, homeostasis, and neurodegeneration. Annu Rev Genet.

[162] Cherry JD, Olschowka JA, O’Banion MK (2014). Neuroinflammation and M2 microglia: the good, the bad, and the inflamed. J Neuroinflamm.

[163] Zhang Z, Zhang Z, Lu H, Yang Q, Wu H, Wang J (2017). Microglial polarization and inflammatory mediators after intracerebral hemorrhage. Mol Neurobiol.

[164] von Maydell D, Jorfi M (2019). The interplay between microglial states and major risk factors in Alzheimer’s disease through the eyes of single-cell RNA-sequencing: beyond black and white. J Neurophysiol.

[165] Keren-Shaul H, Spinrad A, Weiner A, Matcovitch-Natan O, Dvir-Szternfeld R, Ulland TK (2017). A unique microglia type associated with restricting development of Alzheimer’s disease. Cell.

[166] Krasemann S, Madore C, Cialic R, Baufeld C, Calcagno N, Fatimy RE (2017). The TREM2-APOE pathway drives the transcriptional phenotype of dysfunctional microglia in neurodegenerative diseases. Immunity.

[167] Felsky D, Roostaei T, Nho K, Risacher SL, Bradshaw EM, Petyuk V (2019). Neuropathological correlates and genetic architecture of microglial activation in elderly human brain. Nat Commun.

[168] Friedman BA, Srinivasan K, Ayalon G, Meilandt WJ, Lin H, Huntley MA (2018). Diverse brain myeloid expression profiles reveal distinct microglial activation states and aspects of Alzheimer’s disease not evident in mouse models. Cell Rep.

[169] Corder EH, Saunders AM, Strittmatter WJ, Schmechel DE, Gaskell PC, Small GW (1993). Gene dose of apolipoprotein E type 4 allele and the risk of Alzheimer’s disease in late onset families. Science.

[170] Paz-y-Miño CA, García-Cárdenas JM, López-Cortés A, Salazar C, Serrano M, Leone PE (2015). Positive association of the cathepsin D Ala224Val gene polymorphism with the risk of Alzheimer’s disease. Am J Medical Sci..

[171] Scacchi R, Gambina G, Broggio E, Moretto G, Ruggeri M, Corbo RM (2004). The H+ allele of the lipoprotein lipase (LPL) HindIII intronic polymorphism and the risk for sporadic late-onset Alzheimer’s disease. Neurosci Lett.

[172] Pottier C, Ravenscroft TA, Brown PH, Finch NA, Baker M, Parsons M (2016). TYROBP genetic variants in early-onset Alzheimer’s disease. Neurobiol Aging.

[173] Keren-Shaul H, Spinrad A, Weiner A, Matcovitch-Natan O, Dvir-Szternfeld R, Ulland TK (2017). A unique microglia type associated with restricting development of Alzheimer’s disease. Cell.

[174] Götzl JK, Brendel M, Werner G, Parhizkar S, Monasor LS, Kleinberger G (2019). Opposite microglial activation stages upon loss of PGRN or TREM2 result in reduced cerebral glucose metabolism. Embo Mol Med.

[175] Clayton K, Delpech JC, Herron S, Iwahara N, Ericsson M, Saito T (2021). Plaque associated microglia hyper-secrete extracellular vesicles and accelerate tau propagation in a humanized APP mouse model. Mol Neurodegener.

[176] Dani M, Wood M, Mizoguchi R, Fan Z, Walker Z, Morgan R (2018). Microglial activation correlates in vivo with both tau and amyloid in Alzheimer’s disease. Brain.

[177] Fan Z, Brooks DJ, Okello A, Edison P (2017). An early and late peak in microglial activation in Alzheimer’s disease trajectory. Brain.

[178] Sarlus H, Heneka MT (2017). Microglia in Alzheimer’s disease. J Clin Invest.

[179] Condello C, Yuan P, Schain A, Grutzendler J (2015). Microglia constitute a barrier that prevents neurotoxic protofibrillar Aβ42 hotspots around plaques. Nat Commun.

[180] Venegas C, Kumar S, Franklin BS, Dierkes T, Brinkschulte R, Tejera D (2017). Microglia-derived ASC specks cross-seed amyloid-β in Alzheimer’s disease. Nature.

[181] Maphis N, Xu G, Kokiko-Cochran ON, Jiang S, Cardona A, Ransohoff RM (2015). Reactive microglia drive tau pathology and contribute to the spreading of pathological tau in the brain. Brain.

[182] Asai H, Ikezu S, Tsunoda S, Medalla M, Luebke J, Haydar T (2015). Depletion of microglia and inhibition of exosome synthesis halt tau propagation. Nat Neurosci.

[183] McGeer PL, McGeer EG (2013). The amyloid cascade-inflammatory hypothesis of Alzheimer disease: implications for therapy. Acta Neuropathol.

[184] Laurent C, Buée L, Blum D (2018). Tau and neuroinflammation: what impact for Alzheimer’s disease and tauopathies?. Biomed J.

[185] Morales I, Jiménez JM, Mancilla M, Maccioni RB (2013). Tau oligomers and fibrils induce activation of microglial cells. J Alzheimer’s Dis.

[186] Sheng JG, Jones RA, Zhou XQ, McGinness JM, Eldik LJV, Mrak RE (2001). Interleukin-1 promotion of MAPK-p38 overexpression in experimental animals and in Alzheimer’s disease: potential significance for tau protein phosphorylation. Neurochem Int.

[187] Lin PB, Tsai AP, Nho K, Lamb BT, Oblak AL (2021). INPP5D regulates the amyloid pathology in Alzheimer’s disease. Alzheimer’s Dementia..

[188] Kosoy R, Fullard JF, Zeng B, Bendl J, Dong P, Rahman S (2022). Genetics of the human microglia regulome refines Alzheimer’s disease risk loci. Nat Genet.

[189] Pimenova AA, Herbinet M, Gupta I, Machlovi SI, Bowles KR, Marcora E (2021). Alzheimer’s-associated PU.1 expression levels regulate microglial inflammatory response. Neurobiol Dis.

[190] Huang K, Marcora E, Pimenova AA, Narzo AFD, Project TIG of A, Initiative TADN (2017). A common haplotype lowers PU.1 expression in myeloid cells and delays onset of Alzheimer’s disease. Nat Neurosci..

[191] Deczkowska A, Matcovitch-Natan O, Tsitsou-Kampeli A, Ben-Hamo S, Dvir-Szternfeld R, Spinrad A (2017). Mef2C restrains microglial inflammatory response and is lost in brain ageing in an IFN-I-dependent manner. Nat Commun.

[192] Xue F, Tian J, Yu C, Du H, Guo L (2021). Type I interferon response-related microglial Mef2c deregulation at the onset of Alzheimer’s pathology in 5×FAD mice. Neurobiol Dis.

[193] Da Mesquita S, Louveau A, Vaccari A, Smirnov I, Cornelison RC, Kingsmore KM (2018). Functional aspects of meningeal lymphatics in ageing and Alzheimer’s disease. Nature.

[194] Louveau A, Herz J, Alme MN, Salvador AF, Dong MQ, Viar KE (2018). CNS lymphatic drainage and neuroinflammation are regulated by meningeal lymphatic vasculature. Nat Neurosci.

[195] Lohela TJ, Lilius TO, Nedergaard M (2022). The glymphatic system: implications for drugs for central nervous system diseases. Nat Rev Drug Discov.

[196] Louveau A, Plog BA, Antila S, Alitalo K, Nedergaard M, Kipnis J (2017). Understanding the functions and relationships of the glymphatic system and meningeal lymphatics. J Clin Invest.

[197] Iliff JJ, Wang M, Liao Y, Plogg BA, Peng W, Gundersen GA (2012). A paravascular pathway facilitates CSF flow through the brain parenchyma and the clearance of interstitial solutes, including amyloid β. Sci Transl Med..

[198] Xie L, Kang H, Xu Q, Chen MJ, Liao Y, Thiyagarajan M, et al. Sleep drives metabolite clearance from the adult brain. Science. 2013;342:373–7. Available from: http://science.sciencemag.org/content/sci/342/6156/373.full.pdf. 10.1126/science.1241224 PMC3880190 24136970

[199] Hablitz LM, Vinitsky HS, Sun Q, Stæger FF, Sigurdsson B, Mortensen KN (2019). Increased glymphatic influx is correlated with high EEG delta power and low heart rate in mice under anesthesia. Sci Adv.

[200] Ringstad G, Valnes LM, Dale AM, Pripp AH, Vatnehol S-AS, Emblem KE (2018). Brain-wide glymphatic enhancement and clearance in humans assessed with MRI. Jci Insight.

[201] Ishida K, Yamada K, Nishiyama R, Hashimoto T, Nishida I, Abe Y (2022). Glymphatic system clears extracellular tau and protects from tau aggregation and neurodegeneration. J Exp Med.

[202] Wang C, Holtzman DM (2020). Bidirectional relationship between sleep and Alzheimer’s disease: role of amyloid, tau, and other factors. Neuropsychopharmacol.

[203] Bah TM, Goodman J, Iliff JJ (2019). Sleep as a Therapeutic Target in the Aging Brain. Neurotherapeutics.

[204] Winer JR, Morehouse A, Fenton L, Harrison TM, Ayangma L, Reed M (2021). Tau and β-amyloid burden predict actigraphy-measured and self-reported impairment and misperception of human sleep. J Neurosci Official J Soc Neurosci.

[205] Ma Q, Ineichen BV, Detmar M, Proulx ST (2017). Outflow of cerebrospinal fluid is predominantly through lymphatic vessels and is reduced in aged mice. Nat Commun.

[206] Mesquita SD, Herz J, Wall M, Dykstra T, de Lima KA, Norris GT (2021). Aging-associated deficit in CCR7 is linked to worsened glymphatic function, cognition, neuroinflammation, and β-amyloid pathology. Sci Adv.

[207] Zenaro E, Pietronigro E, Bianca VD, Piacentino G, Marongiu L, Budui S (2015). Neutrophils promote Alzheimer’s disease–like pathology and cognitive decline via LFA-1 integrin. Nat Med.

[208] Malm TM, Koistinaho M, Pärepalo M, Vatanen T, Ooka A, Karlsson S (2005). Bone-marrow-derived cells contribute to the recruitment of microglial cells in response to β-amyloid deposition in APP/PS1 double transgenic Alzheimer mice. Neurobiol Dis.

[209] Simard AR, Soulet D, Gowing G, Julien J-P, Rivest S (2006). Bone marrow-derived microglia play a critical role in restricting senile plaque formation in Alzheimer’s disease. Neuron.

[210] Town T, Laouar Y, Pittenger C, Mori T, Szekely CA, Tan J (2008). Blocking TGF-β–Smad2/3 innate immune signaling mitigates Alzheimer-like pathology. Nat Med.

[211] Michaud J-P, Bellavance M-A, Préfontaine P, Rivest S (2013). Real-time in vivo imaging reveals the ability of monocytes to clear vascular amyloid beta. Cell Rep.

[212] Baruch K, Deczkowska A, Rosenzweig N, Tsitsou-Kampeli A, Sharif AM, Matcovitch-Natan O (2016). PD-1 immune checkpoint blockade reduces pathology and improves memory in mouse models of Alzheimer’s disease. Nat Med.

[213] Rogers J, Luber-Narod J, Styren SD, Civin WH (1988). Expression of immune system-associated antigens by cells of the human central nervous system: relationship to the pathology of Alzheimer’s disease. Neurobiol Aging.

[214] Togo T, Akiyama H, Iseki E, Kondo H, Ikeda K, Kato M (2002). Occurrence of T cells in the brain of Alzheimer’s disease and other neurological diseases. J Neuroimmunol.

[215] Merlini M, Kirabali T, Kulic L, Nitsch RM, Ferretti MT (2018). Extravascular CD3+ T cells in brains of Alzheimer disease patients correlate with Tau but not with amyloid pathology: an immunohistochemical study. Neurodegener Dis.

[216] Unger MS, Li E, Scharnagl L, Poupardin R, Altendorfer B, Mrowetz H (2020). CD8+ T-cells infiltrate Alzheimer’s disease brains and regulate neuronal- and synapse-related gene expression in APP-PS1 transgenic mice. Brain Behav Immun.

[217] Monsonego A, Imitola J, Petrovic S, Zota V, Nemirovsky A, Baron R (2006). Aβ-induced meningoencephalitis is IFN-γ-dependent and is associated with T cell-dependent clearance of Aβ in a mouse model of Alzheimer’s disease. P Natl Acad Sci USA.

[218] Ferretti MT, Merlini M, Späni C, Gericke C, Schweizer N, Enzmann G (2016). T-cell brain infiltration and immature antigen-presenting cells in transgenic models of Alzheimer’s disease-like cerebral amyloidosis. Brain Behav Immun.

[219] Browne TC, McQuillan K, McManus RM, O’Reilly J-A, Mills KHG, Lynch MA (2013). IFN-γ production by amyloid β–specific Th1 cells promotes microglial activation and increases plaque burden in a mouse model of Alzheimer’s disease. J Immunol.

[220] McManus RM, Finucane OM, Wilk MM, Mills KHG, Lynch MA (2017). FTY720 attenuates infection-induced enhancement of Aβ accumulation in APP/PS1 mice by modulating astrocytic activation. J Neuroimmune Pharm.

[221] MacPherson KP, Sompol P, Kannarkat GT, Chang J, Sniffen L, Wildner ME (2017). Peripheral administration of the soluble TNF inhibitor XPro1595 modifies brain immune cell profiles, decreases beta-amyloid plaque load, and rescues impaired long-term potentiation in 5xFAD mice. Neurobiol Dis.

[222] Shukla AK, McIntyre LL, Marsh SE, Schneider CA, Hoover EM, Walsh CM (2019). CD11a expression distinguishes infiltrating myeloid cells from plaque-associated microglia in Alzheimer’s disease. Glia.

[223] Mittal K, Eremenko E, Berner O, Elyahu Y, Strominger I, Apelblat D (2019). CD4 T cells induce a subset of MHCII-expressing microglia that attenuates Alzheimer pathology. Iscience.

[224] Machhi J, Yeapuri P, Lu Y, Foster E, Chikhale R, Herskovitz J (2021). CD4+ effector T cells accelerate Alzheimer’s disease in mice. J Neuroinflamm.

[225] Zhang J, Ke K-F, Liu Z, Qiu Y-H, Peng Y-P (2013). Th17 cell-mediated neuroinflammation is involved in neurodegeneration of Aβ1-42-induced Alzheimer’s disease model rats. PLoS ONE.

[226] Pietronigro E, Zenaro E, Bianca VD, Dusi S, Terrabuio E, Iannoto G (2019). Blockade of α4 integrins reduces leukocyte–endothelial interactions in cerebral vessels and improves memory in a mouse model of Alzheimer’s disease. Sci Rep-uk.

[227] Baruch K, Rosenzweig N, Kertser A, Deczkowska A, Sharif AM, Spinrad A (2015). Breaking immune tolerance by targeting Foxp3+ regulatory T cells mitigates Alzheimer’s disease pathology. Nat Commun.

[228] Dansokho C, Ahmed DA, Aid S, Toly-Ndour C, Chaigneau T, Calle V (2016). Regulatory T cells delay disease progression in Alzheimer-like pathology. Brain.

[229] Kim K, Wang X, Ragonnaud E, Bodogai M, Illouz T, DeLuca M (2021). Therapeutic B-cell depletion reverses progression of Alzheimer’s disease. Nat Commun.

[230] Banks WA, Reed MJ, Logsdon AF, Rhea EM, Erickson MA (2021). Healthy aging and the blood–brain barrier. Nat Aging.

[231] Schaeffer S, Iadecola C (2021). Revisiting the neurovascular unit. Nat Neurosci.

[232] Deane R, Zlokovic BV (2007). Role of the blood-brain barrier in the pathogenesis of Alzheimer’s disease. Curr Alzheimer Res..

[233] Erickson MA, Banks WA (2013). Blood-brain barrier dysfunction as a cause and consequence of Alzheimer’s disease. J Cereb Blood Flow Metabolism.

[234] Iadecola C (2017). The neurovascular unit coming of age: a journey through neurovascular coupling in health and disease. Neuron.

[235] Montagne A, Barnes SR, Sweeney MD, Halliday MR, Sagare AP, Zhao Z, Toga AW, Jacobs RE, Liu CY, Amezcua L, Harrington MG (2015). Blood-brain barrier breakdown in the aging human hippocampus. Neuron.

[236] Sweeney MD, Montagne A, Sagare AP, Nation DA, Schneider LS, Chui HC (2019). Vascular dysfunction—the disregarded partner of Alzheimer’s disease. Alzheimer’s Dementia.

[237] Halliday MR, Rege SV, Ma Q, Zhao Z, Miller CA, Winkler EA (2015). Accelerated pericyte degeneration and blood–brain barrier breakdown in apolipoprotein E4 carriers with Alzheimer’s disease. J Cereb Blood Flow Metabolism..

[238] Janelidze S, Hertze J, Nägga K, Nilsson K, Nilsson C, Group the SBS (2017). Increased blood-brain barrier permeability is associated with dementia and diabetes but not amyloid pathology or APOE genotype. Neurobiol Aging..

[239] Nation DA, Sweeney MD, Montagne A, Sagare AP, D’Orazio LM, Pachicano M (2019). Blood–brain barrier breakdown is an early biomarker of human cognitive dysfunction. Nat Med.

[240] Sengillo JD, Winkler EA, Walker CT, Sullivan JS, Johnson M, Zlokovic BV (2013). Pericytes in Alzheimer’s disease. Brain Pathol.

[241] Haar HJ, Jansen JFA, Jeukens CRLPN, Burgmans S, Buchem MA, Muller M (2017). Subtle blood-brain barrier leakage rate and spatial extent: considerations for dynamic contrast-enhanced MRI. Med Phys.

[242] van de Haar HJ, Jansen JFA, van Osch MJP, van Buchem MA, Muller M, Wong SM (2016). Neurovascular unit impairment in early Alzheimer’s disease measured with magnetic resonance imaging. Neurobiol Aging.

[243] Baloyannis SJ, Baloyannis IS (2012). The vascular factor in Alzheimer’s disease: a study in Golgi technique and electron microscopy. J Neurol Sci.

[244] Ma Q, Zhao Z, Sagare AP, Wu Y, Wang M, Owens NC (2018). Blood-brain barrier-associated pericytes internalize and clear aggregated amyloid-β42 by LRP1-dependent apolipoprotein E isoform-specific mechanism. Mol Neurodegener.

[245] Halliday R, Rege V, Ma Q, Zhao Z, Miller A, Winkler A (2016). Accelerated pericyte degeneration and blood-brain barrier breakdown in apolipoprotein E4 carriers with Alzheimer’s disease. J Cereb Blood Flow Metab.

[246] Janelidze S, Hertze J, Nagga K, Nilsson K, Nilsson C, Study GSBF (2017). Increased blood-brain barrier permeability is associated with dementia and diabetes but not amyloid pathology or APOE genotype. Neurobiol Aging..

[247] Ma Q, Zhao Z, Sagare P, Wu Y, Wang M, Owens C (2018). Blood-brain barrier-associated pericytes internalize and clear aggregated amyloid-beta42 by LRP1-dependent apolipoprotein E isoform-specific mechanism. Mol Neurodegener.

[248] Miners S, Schulz I, Love S (2018). Differing associations between Abeta accumulation, hypoperfusion, blood-brain barrier dysfunction and loss of PDGFRB pericyte marker in the precuneus and parietal white matter in Alzheimer’s disease. J Cereb Blood Flow Metab.

[249] Sengillo D, Winkler A, Walker T, Sullivan S, Johnson M, Zlokovic V (2013). Deficiency in mural vascular cells coincides with blood-brain barrier disruption in Alzheimer’s disease. Brain Pathol.

[250] van de Haar HJ, Jansen JFA, Jeukens CRLPN, Burgmans S, van Buchem MA, Muller M (2017). Subtle blood-brain barrier leakage rate and spatial extent: considerations for dynamic contrast-enhanced MRI. Med Phys.

[251] van de Haar HJ, Jansen JF, van Osch MJ, van Buchem MA, Muller M, Wong SM, Hofman PA, Burgmans S, Verhey FR, Backes WH (2016). Neurovascular unit impairment in early Alzheimer’s disease measured with magnetic resonance imaging. Neurobiol Aging.

[252] Kapasi A, Leurgans SE, Arvanitakis Z, Barnes LL, Bennett DA, Schneider JA (2021). Aβ (amyloid beta) and Tau tangle pathology modifies the association between small vessel disease and cortical microinfarcts. Stroke.

[253] van de Haar HJ, Burgmans S, Jansen JFA, van Osch MJP, van Buchem MA, Muller M (2016). Blood-brain barrier leakage in patients with early Alzheimer disease. Radiology.

[254] van de Haar H, Jansen JFA, van Osch MJP, van Buchem MA, Backes WH (2016). Neurovascular unit impairment in early Alzheimer’s disease measured with magnetic resonance imaging. Neurobiol Aging.

[255] Sweeney MD, Zhao Z, Montagne A, Nelson AR, Zlokovic BV (2019). Blood-brain barrier: from physiology to disease and back. Physiol Rev.

[256] Mason HD, McGavern DB (2022). How the immune system shapes neurodegenerative diseases. Trends Neurosci.

[257] Sommer A, Winner B, Prots I (2017). The Trojan horse - neuroinflammatory impact of T cells in neurodegenerative diseases. Mol Neurodegener.

[258] Marchetti L, Engelhardt B. Immune cell trafficking across the blood-brain barrier in the absence and presence of neuroinflammation. Vasc Biol. 2020;2:H1-18. Available from: https://www.ncbi.nlm.nih.gov/pubmed/32923970. 10.1530/VB-19-0033 PMC7439848 32923970

[259] Wilson EH, Weninger W, Hunter CA (2010). Trafficking of immune cells in the central nervous system. J Clin Invest.

[260] Baik SH, Cha MY, Hyun YM, Cho H, Hamza B, Kim DK (2014). Migration of neutrophils targeting amyloid plaques in Alzheimer’s disease mouse model. Neurobiol Aging.

[261] Rossi B, Santos-Lima B, Terrabuio E, Zenaro E, Constantin G (2021). Common peripheral immunity mechanisms in multiple sclerosis and Alzheimer’s disease. Front Immunol.

[262] Kuyumcu ME, Yesil Y, Oztürk ZA, Kizilarslanoğlu C, Etgül S, Halil M (2012). The evaluation of neutrophil-lymphocyte ratio in Alzheimer’s disease. Dement Geriatr Cogn.

[263] Dong X, Nao J, Shi J, Zheng D (2019). Predictive value of routine peripheral blood biomarkers in Alzheimer’s disease. Front Aging Neurosci..

[264] Kebir H, Kreymborg K, Ifergan I, Dodelet-Devillers A, Cayrol R, Bernard M (2007). Human TH17 lymphocytes promote blood-brain barrier disruption and central nervous system inflammation. Nat Med.

[265] Sas AR, Carbajal KS, Jerome AD, Menon R, Yoon C, Kalinski AL (2020). A new neutrophil subset promotes CNS neuron survival and axon regeneration. Nat Immunol.

[266] Naert G, Rivest S (2011). CC Chemokine receptor 2 deficiency aggravates cognitive impairments and amyloid pathology in a transgenic mouse model of Alzheimer’s disease. J Neurosci.

[267] Khoury JE, Toft M, Hickman SE, Means TK, Terada K, Geula C (2007). Ccr2 deficiency impairs microglial accumulation and accelerates progression of Alzheimer-like disease. Nat Med.

[268] Fiala M, Lin J, Ringman J, Kermani-Arab V, Tsao G, Patel A (2005). Ineffective phagocytosis of amyloid-β by macrophages of Alzheimer’s disease patients. J Alzheimer’s Dis.

[269] Monsonego A, Nemirovsky A, Harpaz I (2013). CD4 T cells in immunity and immunotherapy of Alzheimer’s disease. Immunology.

[270] Doecke JD, Laws SM, Faux NG, Wilson W, Burnham SC, Lam C-P (2012). Blood-based protein biomarkers for diagnosis of Alzheimer disease. Arch Neurol-chicago..

[271] Chen J-M, Jiang G-X, Li Q-W, Zhou Z-M, Cheng Q (2014). Increased serum levels of interleukin-18, -23 and -17 in Chinese patients with Alzheimer’s disease. Dement Geriatr Cogn.

[272] Durelli L, Conti L, Clerico M, Boselli D, Contessa G, Ripellino P (2009). T-helper 17 cells expand in multiple sclerosis and are inhibited by interferon-β. Ann Neurol.

[273] Brucklacher-Waldert V, Stuerner K, Kolster M, Wolthausen J, Tolosa E (2009). Phenotypical and functional characterization of T helper 17 cells in multiple sclerosis. Brain.

[274] Ashtari F, Madanian R, Shaygannejad V, Zarkesh SH, Ghadimi K (2019). Serum levels of IL-6 and IL-17 in multiple sclerosis, neuromyelitis optica patients and healthy subjects. Int J Physiology Pathophysiol Pharmacol.

[275] Pellicanò M, Larbi A, Goldeck D, Colonna-Romano G, Buffa S, Bulati M (2012). Immune profiling of Alzheimer patients. J Neuroimmunol.

[276] Schetters STT, Gomez-Nicola D, Garcia-Vallejo JJ, Kooyk YV (2018). Neuroinflammation: microglia and T cells get ready to tango. Front Immunol.

[277] Goddery EN, Fain CE, Lipovsky CG, Ayasoufi K, Yokanovich LT, Malo CS (2021). Microglia and perivascular macrophages act as antigen presenting cells to promote CD8 T cell infiltration of the brain. Front Immunol.

[278] Reagin KL, Funk KE (2022). The role of antiviral CD8+ T cells in cognitive impairment. Curr Opin Neurobiol.

[279] Batterman KV, Cabrera PE, Moore TL, Rosene DL (2021). T cells actively infiltrate the white matter of the aging monkey brain in relation to increased microglial reactivity and cognitive decline. Front Immunol.

[280] Stojić-Vukanić Z, Hadžibegović S, Nicole O, Nacka-Aleksić M, Leštarević S, Leposavić G (2020). CD8+ T cell-mediated mechanisms contribute to the progression of neurocognitive impairment in both multiple sclerosis and Alzheimer’s disease?. Front Immunol.

[281] Cencioni MT, Mattoscio M, Magliozzi R, Bar-Or A, Muraro PA (2021). B cells in multiple sclerosis — from targeted depletion to immune reconstitution therapies. Nat Rev Neurol.

[282] Myhr K-M, Torkildsen Ø, Lossius A, Bø L, Holmøy T (2019). B cell depletion in the treatment of multiple sclerosis. Expert Opin Biol Th.

[283] Yednock TA, Cannon C, Fritz LC, Sanchez-Madrid F, Steinman L, Karin N (1992). Prevention of experimental autoimmune encephalomyelitis by antibodies against α4βl integrin. Nature.

[284] Polman CH, O’Connor PW, Havrdova E, Hutchinson M, Kappos L, Miller DH (2006). A randomized, placebo-controlled trial of natalizumab for relapsing multiple sclerosis. New Engl J Med.

[285] Bierhansl L, Hartung H-P, Aktas O, Ruck T, Roden M, Meuth SG. Thinking outside the box: non-canonical targets in multiple sclerosis. Nat Rev Drug Discov. 2022;21(8):578–600. 10.1038/s41573-022-00477-5 PMC9169033 35668103

[286] Wang J, Gu BJ, Masters CL, Wang Y-J (2017). A systemic view of Alzheimer disease — insights from amyloid-β metabolism beyond the brain. Nat Rev Neurol.

[287] Holmes C, El-Okl M, Williams AL, Cunningham C, Wilcockson D, Perry VH (2003). Systemic infection, interleukin 1β, and cognitive decline in Alzheimer’s disease. J Neurology Neurosurg Psychiatry..

[288] Heneka MT, Carson MJ, Khoury JE, Landreth GE, Brosseron F, Feinstein DL (2015). Neuroinflammation in Alzheimer’s disease. Lancet Neurol.

[289] Holmes C, Cunningham C, Zotova E, Culliford D, Perry VH (2011). Proinflammatory cytokines, sickness behavior, and Alzheimer disease. Neurology.

[290] Wood LB, Winslow AR, Proctor EA, McGuone D, Mordes DA, Frosch MP (2015). Identification of neurotoxic cytokines by profiling Alzheimer’s disease tissues and neuron culture viability screening. Sci Rep-uk..

[291] vom Berg J, Prokop S, Miller KR, Obst J, Kälin RE, Lopategui-Cabezas I (2012). Inhibition of IL-12/IL-23 signaling reduces Alzheimer’s disease–like pathology and cognitive decline. Nat Med.

[292] Moir RD, Lathe R, Tanzi RE (2018). The antimicrobial protection hypothesis of Alzheimer’s disease. Alzheimer’s Dementia..

[293] Bukhbinder AS, Ling Y, Hasan O, Jiang X, Kim Y, Phelps KN, et al. Risk of Alzheimer’s disease following influenza vaccination: a claims-based cohort study using propensity score matching. J Alzheimer’s Dis. 2022;88(3):1061–74. 10.3233/JAD-220361 PMC9484126 35723106

[294] Klein BY, Greenblatt CL, Gofrit ON, Bercovier H (2022). Bacillus Calmette-Guérin in immuno-regulation of Alzheimer’s disease. Front Aging Neurosci.

[295] Amran A, Lin Y, Kim Y, Bernstam E, Jiang X, Schulz PE. Influenza vaccination is associated with a reduced incidence of Alzheimer’s disease. Alzheimer’s Dementia. 2020;16:e041693.

[296] Kim JI, Zhu D, Barry E, Kovac E, Aboumohamed A, Agalliu I (2021). Intravesical Bacillus Calmette-Guérin treatment is inversely associated with the risk of developing Alzheimer disease or other dementia among patients with non–muscle-invasive bladder cancer. Clin Genitourin Canc.

[297] Klinger D, Hill BL, Barda N, Halperin E, Gofrit ON, Greenblatt CL (2021). Bladder cancer immunotherapy by BCG is associated with a significantly reduced risk of Alzheimer’s disease and Parkinson’s disease. Nato Adv Sci Inst Se.

[298] Verreault R, Laurin D, Lindsay J, Serres GD (2001). Past exposure to vaccines and subsequent risk of Alzheimer’s disease. Cmaj Can Medical Assoc J J De L’association Medicale Can.

[299] Holmes C, Cunningham C, Zotova E, Woolford J, Dean C, Kerr S (2009). Systemic inflammation and disease progression in Alzheimer disease. Neurology.

[300] Sipilä PN, Heikkilä N, Lindbohm JV, Hakulinen C, Vahtera J, Elovainio M (2021). Hospital-treated infectious diseases and the risk of dementia: a large, multicohort, observational study with a replication cohort. Lancet Infect Dis.

[301] Eriksson LI, Lundholm C, Narasimhalu K, Sandin R, Jin Y, Gatz M (2019). Hospitalization, surgery, and incident dementia. Alzheimer’s Dementia..

[302] Phelan EA, Borson S, Grothaus L, Balch S, Larson EB (2012). Association of incident dementia with hospitalizations. JAMA.

[303] Pandharipande PP, Girard TD, Jackson JC, Morandi A, Thompson JL, Pun BT (2013). Long-term cognitive impairment after critical illness. New Engl J Med.

[304] Alexander GC, Knopman DS, Emerson SS, Ovbiagele B, Kryscio RJ, Perlmutter JS (2021). Revisiting FDA Approval of Aducanumab. New Engl J Med..

[305] Rabinovici GD (2021). Controversy and progress in Alzheimer’s disease — FDA approval of aducanumab. New Engl J Med..

[306] Moir RD, Tseitlin KA, Soscia S, Hyman BT, Irizarry MC, Tanzi RE (2005). Autoantibodies to Redox-modified oligomeric Aβ are attenuated in the plasma of Alzheimer’s disease patients*. J Biol Chem.

[307] Rynearson KD, Ponnusamy M, Prikhodko O, Xie Y, Zhang C, Nguyen P (2021). Preclinical validation of a potent γ-secretase modulator for Alzheimer’s disease prevention. J Exp Med.

[308] McDade E, Llibre-Guerra JJ, Holtzman DM, Morris JC, Bateman RJ (2021). The informed road map to prevention of Alzheimer disease: a call to arms. Mol Neurodegener.

[309] Tanzi RE (2021). FDA approval of Aduhelm paves a new path for Alzheimer’s disease. Acs Chem Neurosci.

[310] Schott JM, Aisen PS, Cummings JL, Howard RJ, Fox NC (2019). Unsuccessful trials of therapies for Alzheimer’s disease. Lancet.

[311] Aisen PS, Jimenez-Maggiora GA, Rafii MS, Walter S, Raman R (2022). Early-stage Alzheimer disease: getting trial-ready. Nat Rev Neurol.

[312] Dyck CH van, Swanson CJ, Aisen P, Bateman RJ, Chen C, Gee M, et al. Lecanemab in Early Alzheimer’s Disease. New Engl J Med. 2023;388(1):9–21. 10.1056/NEJMoa2212948 36449413

[313] Rofo F, Meier SR, Metzendorf NG, Morrison JI, Petrovic A, Syvänen S (2022). A brain-targeting bispecific-multivalent antibody clears soluble amyloid-beta aggregates in Alzheimer’s disease mice. Neurotherapeutics.

[314] Network DIA, Ringman JM, Goate A, Masters CL, Cairns NJ, Danek A (2014). Genetic heterogeneity in Alzheimer disease and implications for treatment strategies. Curr Neurol Neurosci.

[315] Devi G, Scheltens P (2018). Heterogeneity of Alzheimer’s disease: consequence for drug trials?. Alzheimer’s Res Ther.

[316] Morenas-Rodríguez E, Li Y, Nuscher B, Franzmeier N, Xiong C, Suárez-Calvet M (2022). Soluble TREM2 in CSF and its association with other biomarkers and cognition in autosomal-dominant Alzheimer’s disease: a longitudinal observational study. Lancet Neurol.

[317] Zhao P, Xu Y, Jiang L, Fan X, Li L, Li X (2022). A tetravalent TREM2 agonistic antibody reduced amyloid pathology in a mouse model of Alzheimer’s disease. Sci Transl Med.

[318] Zhao N, Qiao W, Li F, Ren Y, Zheng J, Martens YA (2022). Elevating microglia TREM2 reduces amyloid seeding and suppresses disease-associated microglia. J Exp Med.

[319] Griciuc A, Federico AN, Natasan J, Forte AM, McGinty D, Nguyen H (2020). Gene therapy for Alzheimer’s disease targeting CD33 reduces amyloid beta accumulation and neuroinflammation. Hum Mol Genet.

[320] Li Z, Shue F, Zhao N, Shinohara M, Bu G (2020). APOE2: protective mechanism and therapeutic implications for Alzheimer’s disease. Mol Neurodegener.

[321] Dodart J-C, Marr RA, Koistinaho M, Gregersen BM, Malkani S, Verma IM (2005). Gene delivery of human apolipoprotein E alters brain Aβ burden in a mouse model of Alzheimer’s disease. Proc National Acad Sci..

[322] Nelson PT, Pious NM, Jicha GA, Wilcock DM, Fardo DW, Estus S (2013). APOE-ε2 and APOE-ε4 correlate with increased amyloid accumulation in cerebral vasculature. J Neuropathology Exp Neurol.

[323] Schilling S, DeStefano AL, Sachdev PS, Choi SH, Mather KA, DeCarli CD (2013). APOE genotype and MRI markers of cerebrovascular disease. Neurology.

[324] Williams T, Borchelt DR, Chakrabarty P (2020). Therapeutic approaches targeting Apolipoprotein E function in Alzheimer’s disease. Mol Neurodegener.

[325] Vogel JW, Hansson O. Subtypes of Alzheimer’s disease: questions, controversy, and meaning. Trends Neurosci. 2022;45(5):342–5. 10.1016/j.tins.2022.02.001 PMC11108088 35227519

[326] Uhr JW (1964). The heterogeneity of the immune response. Science.

[327] Satija R, Shalek AK (2014). Heterogeneity in immune responses: from populations to single cells. Trends Immunol.

[328] Walker LC (2016). Proteopathic strains and the heterogeneity of neurodegenerative diseases. Annu Rev Genet.

[329] Gause WC, Rothlin C, Loke P (2020). Heterogeneity in the initiation, development and function of type 2 immunity. Nat Rev Immunol.

[330] Jakobsson HE, Abrahamsson TR, Jenmalm MC, Harris K, Quince C, Jernberg C (2014). Decreased gut microbiota diversity, delayed Bacteroidetes colonisation and reduced Th1 responses in infants delivered by Caesarean section. Gut.

[331] Nabhani ZA, Dulauroy S, Marques R, Cousu C, Bounny SA, Déjardin F (2019). A weaning reaction to microbiota is required for resistance to immunopathologies in the adult. Immunity.

[332] Wu K-M, Zhang Y-R, Huang Y-Y, Dong Q, Tan L, Yu J-T (2021). The role of the immune system in Alzheimer’s disease. Ageing Res Rev.

[333] Weiner HL, Frenkel D (2006). Immunology and immunotherapy of Alzheimer’s disease. Nat Rev Immunol.

[334] Carrasco E, de Heras MMGL, Gabande-Rodriguez E, Desdin-Mico G, Aranda JF, Mittelbrunn M (2021). The role of T cells in age-related diseases. Nat Rev Immunol.

[335] Liu Z, Qiu A-W, Huang Y, Yang Y, Chen J-N, Gu T-T (2019). IL-17A exacerbates neuroinflammation and neurodegeneration by activating microglia in rodent models of Parkinson’s disease. Brain Behav Immun.

[336] Cristiano C, Volpicelli F, Lippiello P, Buono B, Raucci F, Piccolo M (2019). Neutralization of IL-17 rescues amyloid-β-induced neuroinflammation and memory impairment. Brit J Pharmacol.

[337] Alves S, Churlaud G, Audrain M, Michaelsen-Preusse K, Fol R, Souchet B (2016). Interleukin-2 improves amyloid pathology, synaptic failure and memory in Alzheimer’s disease mice. Brain.

[338] Yshii L, Pasciuto E, Bielefeld P, Mascali L, Lemaitre P, Marino M (2022). Astrocyte-targeted gene delivery of interleukin 2 specifically increases brain-resident regulatory T cell numbers and protects against pathological neuroinflammation. Nat Immunol.

[339] Alpert A, Pickman Y, Leipold M, Rosenberg-Hasson Y, Ji X, Gaujoux R (2019). A clinically meaningful metric of immune age derived from high-dimensional longitudinal monitoring. Nat Med.

[340] Ferretti MT, Iulita MF, Cavedo E, Chiesa PA, Dimech AS, Chadha AS (2018). Sex differences in Alzheimer disease — the gateway to precision medicine. Nat Rev Neurol.

[341] Gómez-Isla T, Frosch MP (2022). Lesions without symptoms: understanding resilience to Alzheimer disease neuropathological changes. Nat Rev Neurol.

